# Isogenic patient-derived organoids reveal early neurodevelopmental defects in spinal muscular atrophy initiation

**DOI:** 10.1016/j.xcrm.2024.101659

**Published:** 2024-07-26

**Authors:** Tobias Grass, Zeynep Dokuzluoglu, Felix Buchner, Ines Rosignol, Joshua Thomas, Antonio Caldarelli, Anna Dalinskaya, Jutta Becker, Fabian Rost, Michele Marass, Brunhilde Wirth, Marc Beyer, Lorenzo Bonaguro, Natalia Rodriguez-Muela

**Affiliations:** 1 German Center for Neurodegenerative Diseases e.V. (DZNE), Dresden, Germany; 2 Technische Universität Dresden (TUD), Center for Regenerative Therapies Dresden, Dresden, Germany; 3 Institute of Human Genetics, University Hospital of Cologne, Cologne, Germany; 4 DRESDEN-concept Genome Center, Technology Platform at the Center for Molecular and Cellular Bioengineering, TUD, Dresden, Germany; 5 Max Planck Institute for Molecular Cell Biology and Genetics, Dresden, Germany; 6 Center for Systems Biology Dresden, Dresden, Germany; 7 Center for Molecular Medicine Cologne, University of Cologne, Cologne, Germany; 8 Center for Rare Diseases, University Hospital of Cologne, Cologne, Germany; 9 Systems Medicine, DZNE, Bonn, Germany; 10 PRECISE Platform for Single Cell Genomics and Epigenomics, DZNE & University of Bonn and West German Genome Center, Bonn, Germany; 11 Immunogenomics & Neurodegeneration, DZNE, Bonn, Germany; 12 Genomics & Immunoregulation, LIMES Institute, University of Bonn, Bonn, Germany

**Keywords:** isogenic SMA model, spinal cord, organoids, neurodevelopmental defects, neuromesodermal progenitors

## Abstract

Whether neurodevelopmental defects underlie postnatal neuronal death in neurodegeneration is an intriguing hypothesis only recently explored. Here, we focus on spinal muscular atrophy (SMA), a neuromuscular disorder caused by reduced survival of motor neuron (SMN) protein levels leading to spinal motor neuron (MN) loss and muscle wasting. Using the first isogenic patient-derived induced pluripotent stem cell (iPSC) model and a spinal cord organoid (SCO) system, we show that SMA SCOs exhibit abnormal morphological development, reduced expression of early neural progenitor markers, and accelerated expression of MN progenitor and MN markers. Longitudinal single-cell RNA sequencing reveals marked defects in neural stem cell specification and fewer MNs, favoring mesodermal progenitors and muscle cells, a bias also seen in early SMA mouse embryos. Surprisingly, *SMN2*-to-*SMN1* conversion does not fully reverse these developmental abnormalities. These suggest that early neurodevelopmental defects may underlie later MN degeneration, indicating that postnatal SMN-increasing interventions might not completely amend SMA pathology in all patients.

## Highlights

• Cohort of isogenic control patient-derived SMA iPSC lines
• Accelerated differentiation of SMA spinal motor neurons
• Neuromesodermal fate commitment defects in SMA spinal cord organoids
• Mesodermal bias of neuromesodermal progenitors in early SMA mouse embryos

## Graphical abstract

**Figure:**
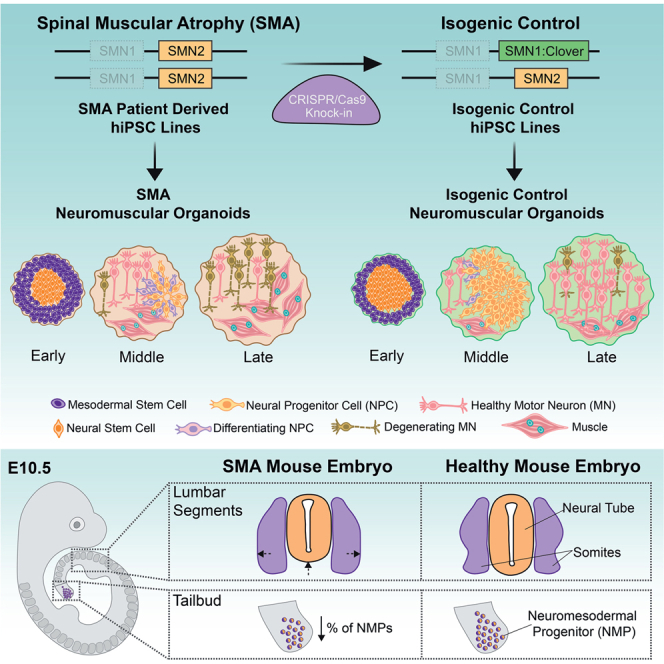

## Teaser

Neurodegenerative diseases are often investigated from a postnatal perspective, ignoring the potential contribution of developmental aspects. Using a spinal cord organoid model derived from isogenic SMA iPSCs, Grass et al. uncover the role of SMN in neuronal progenitor and mesodermal specification. These developmental impairments precede the neuronal loss characteristic of SMA.

## Introduction

Accumulating evidence suggests that neurodegenerative diseases (NDs) may have a developmental component crucial for postmitotic neurons to manifest disease hallmarks. Despite known gene mutations or deletions causing diseases like familial amyotrophic lateral sclerosis, Alzheimer’s, Parkinson’s, or Huntington’s disease, pathology often appears only decades after birth, which has led to a lack of developmental studies on these diseases. However, recent evidence hints at neurodevelopmental alterations that could revolutionize how these NDs are studied, diagnosed, and treated.

Spinal muscular atrophy (SMA) is an autosomal, recessive neuromuscular disease where spinal motor neurons (MNs) degenerate, leading to muscle wasting and, in severe cases, premature death. It is caused by mutations or deletions in the *SMN1* gene, coding for the survival of MN (SMN) protein.^1^^,^^2^ Humans also have a second paralogous gene, *SMN2*, which partially compensates for *SMN1* loss. *SMN2* is almost identical to *SMN1* but carries a critical nucleotide change in exon 7 that disrupts its splicing.^3^ Consequently, only a small fraction of the *SMN2* transcripts produce full-length SMN protein while most *SMN2* transcripts produce truncated, unstable protein.^4^^,^^5^ The SMN2 copy number varies between individuals, and therefore the amount of SMN2-derived full-length SMN protein does too, which greatly accounts for a wide spectrum of SMA disease severities.^6^^,^^7^ SMN is vital for spliceosomal small nuclear ribonucleoproteins (snRNPs) assembly, mediating pre-mRNA splicing.^8^ Complete SMN absence leads to cell death,^9^^,^^10^ and *SMN* knockout mice die at the morula stage.^11^ SMN has other roles, such as in the axonal transport of mRNAs and ribonucleoproteins, ribosomal dynamics, translation,^12^ mitochondrial trafficking,^13^^,^^14^ endosomal and membrane recycling pathways,^15^^,^^16^^,^^17^ and autophagy.^18^^,^^19^ Anomalies in these pathways due to SMN deficiency may cause neural progenitor defects leading to neuron degeneration later in life. While mild SMA types III and IV display classical ND features, severe types 0 and I show developmental disease characteristics,^20^ such as immature motor axons in SMA fetuses and mouse embryos that fail to reach proper radial growth and myelination during embryogenesis.^21^ Depleting SMN in Olig2+ MN progenitors causes an SMA-like phenotype in mice,^22^ and altering SMN levels in neuroblasts affects locomotor function in *Drosophila*.^23^ Further, SMN levels are higher and more essential during prenatal development than postnatally,^24^^,^^25^^,^^26^^,^^27^^,^^28^ making early therapeutic interventions crucial for better outcomes.^29^^,^^30^^,^^31^

Patient-derived *in vitro* models have advanced human neurodevelopment and disease research.^32^^,^^33^ Cerebral organoids and assembloids enhance our understanding of human physiology and pathology and translate well to clinical trials, aiding in discovering and testing new therapies. While extensively used for studying many NDs,^34^ SMA research with these models is still nascent.^35^ A significant limitation in human-based research is the lack of isogenic context in many studies. Healthy control and diseased human induced pluripotent stem cells (hiPSCs) come from different donors with varying genetic backgrounds, only allowing correlative data. To assess genetic differences’ impact on disease phenotypes, targeted mutations in an isogenic context are necessary. Different cell line backgrounds have affected hiPSC-based experiments’ reproducibility and could lead to misleading findings.^36^

This study aimed to generate an SMA spinal cord organoid (SCO) model using isogenic and patient-derived cells to examine the hypothesis of an early developmental alteration underlying the selective postmitotic MN death. We utilized three hiPSC lines from patients with SMA types I, II, and III,^37^ and using a knockin CRISPR-Cas9 approach, we corrected the nucleotide change responsible for the exon 7 skipping^3^ in at least one *SMN2* copy in each of them. We investigated SMN’s functional role in early development, finding that SMA SCOs exhibited faulty and delayed growth compared to their isogenic or healthy counterparts, with defective neural stem cell (NSC) and progenitor cell specification. Longitudinal single-cell RNA sequencing (scRNA-seq) and targeted transcriptomic analysis indicated differential cell distributions in SMA SCOs, with a bias of neuromesodermal progenitors (NMPs) toward muscle cell identity. This impaired neuronal specification could sensitize specific MN populations to degeneration as the disease progresses. Interestingly, developmental abnormalities in type I SCOs were only partially corrected in the isogenic clones. Aligning with these findings, the neural tube area relative to surrounding mesodermal tissue in E10.5 SMA embryos was significantly reduced, and the number of NMPs in the tail bud of the developing spinal cord was also significantly lower compared to control littermates. Overall, our new platform indicates an early developmental role for SMN, suggesting that postnatal SMN restoration might not fully correct pathological phenotypes in all patients with SMA. This platform also provides a precise system to functionally investigate alternative molecular pathways involved in SMA genetics and pathology.

## Results

### Generation of isogenic control hiPSCs from various severities of SMA hiPSC lines

**Figure 1:**
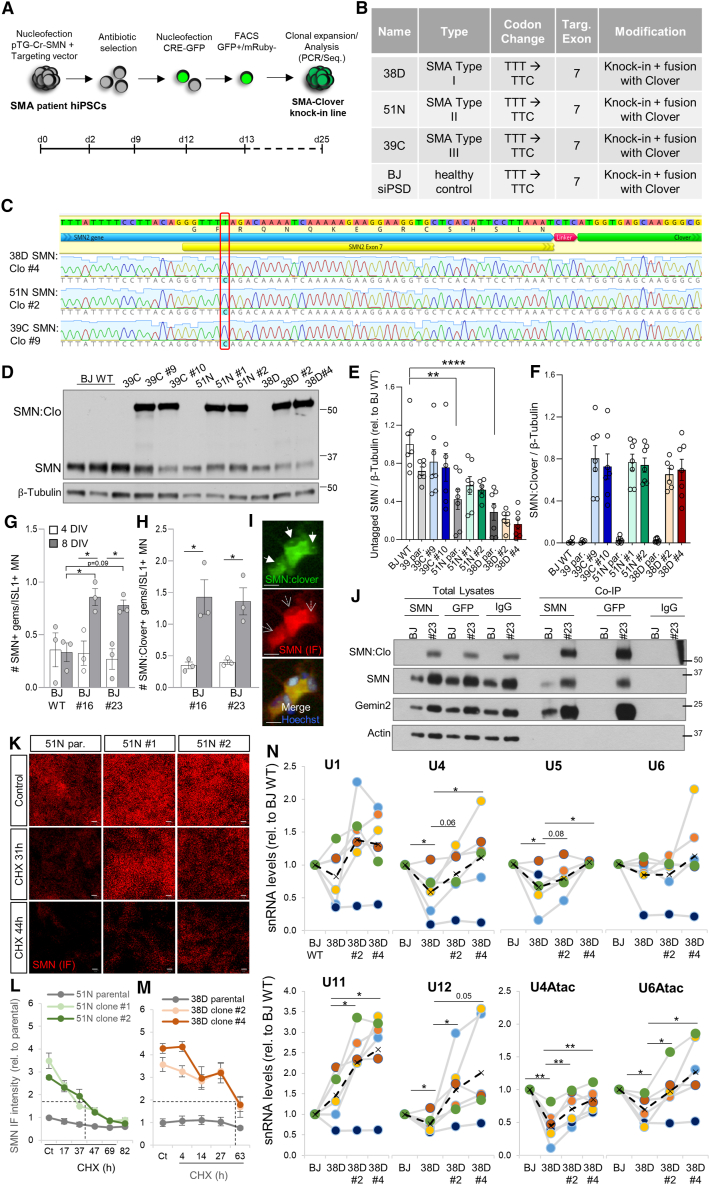
Generation of isogenic control hiPSCs from various severities of SMA hiPSC lines (A) Knockin CRISPR-Cas9-mediated mutagenesis workflow to correct the C-to-T nucleotide change in *SMN2* in SMA hiPSC lines. (B) Isogenic corrected hiPSC lines generated and used for the study. (C) Sanger sequencing results from the successfully targeted SMA lines aligned to exon 7 of *SMN2* gene. Chromatograms from one clone of each successfully targeted SMA line highlighting the corrected C in position 6 of exon 7. See also Figure S1. (D–F) (D) Representative western blot from hiPSC lysates and respective quantifications (E and F) (*N* = 4). (G–I) (G) Quantification of the number of nuclear gems immunostained with anti-SMN antibody or SMN:Clover endogenously labeled (H) in BJ WT and BJ SMN:Clover (#16 and #23) derived MN cultures 4 and 8 days after plating (*N* = 3). Representative SMN immunostained (empty arrows) and SMN:Clover+ (filled arrows) nuclear gems in MNs. Scale bar, 10 μm (I). See also Figures S2A–S2D. (J) Representative immunoprecipitation from BJ WT and BJ SMN:Clover #23 hiPSC lysates. (K–M) (K) Representative SMN-immunostained isogenic SMA type II hiPSC trio treated with CHX. Scale bar, 50 μm. Total SMN protein level quantification upon CHX treatment in the SMA 51N-II (L) and 38D-I (M) hiPSC trios (*N* = 4). See also Figures S2E–S2Y. (N) qPCR analysis of snRNAs from EBs derived from BJ WT and the SMA type I isogenic trio differentiated as in (G)–(I). RNA levels are expressed relative to BJ WT levels for each individual experiment (color-coded; *N* = 5). The dotted line indicates the average values for all experiments. See also Figure S3. One-way ANOVA with Tukey’s (E–H) and Fischer’s LSD (N) multiple comparison test used for statistical analysis.

In most patients with SMA, both *SMN1* alleles are deleted or severely mutated, with less than 5% of cases caused by single point mutations.^20^ Therefore, base-editing or CRISPR-Cas-based approaches targeting *SMN1* are often not possible. For our study, we used three previously described human SMA induced pluripotent stem cell lines,^18^^,^^37^^,^^38^ where no *SMN1* allele was detected by MLPA (multiplex ligation-dependent probe amplification) (Table S1). We employed a CRISPR-Cas9-mediated genome editing approach to generate three isogenic hiPSC trios by targeting the *SMN2* gene (Table S2). For each SMA parental line, two independent isogenic clones were generated by correcting the C-to-T nucleotide change in exon 7 of at least one *SMN2* copy. We edited one SMA type I line (38D-I), one type II line (51N-II), and one type III line (39C-III) using a two-vector knockin targeting approach^39^ (Figures 1A and 1B). Two donor DNA fragments were used: one homologous to intron 6-exon 7 with the corrected exon 7 nucleotide change, and the second homologous to the region downstream of exon 7 containing the coding sequence of the reporter Clover^40^ (Figure S1A). PCR on cDNA from parental and genome-edited lines confirmed the expected size band (∼1,156 bp) (Figure S1B), and Sanger sequencing confirmed the correct *SMN1* edited sequence. MLPA confirmed the absence of *SMN1* and the presence of 2 or 3 *SMN2* copies in the parental SMA lines but failed to identify the converted *SMN2* in isogenic hiPSC clones due to the inability of the probe to bind after *SMN2* conversion to *SMN1* (Table S2). Instead, Sanger sequencing confirmed correct donor DNA integration, converting at least one *SMN2* gene to *SMN1* (Figures 1C, S1E, and S1F). PCR showed at least one untargeted *SMN2* copy in each line (Figures S1C, S1D, and Table S2), indicating a heterozygous *SMN2*-to-*SMN1* conversion. Two clones per line were selected for subsequent studies, all karyotypically normal with no abnormality detected by SNP/copy-number analysis (data not shown) and expressing normal levels of pluripotency markers (OCT4, SOX2, NANOG, and TRA160) (Figure S1G).

We previously showed that SMN protein levels in spinal MN lysates from these SMA hiPSCs are ∼10% for type I and ∼40% for types II and III compared to healthy controls.^37^ Western blot analysis confirmed only untagged SMN protein in parental lines and high levels of SMN:Clover in the corrected clones (Figures 1D–1F). Higher SMN levels in the corrected clones compared to endogenous SMN in the BJ wild-type (WT) line can be attributed to the higher stability of GFP protein variants like Clover in the downstream processing of the protein lysates. We generated an isogenic model to demonstrate causality, as comparing disease lines with “healthy” ones having different genetic backgrounds is insufficient to demonstrate causality. However, as the SMA hiPSC lines used in this study, despite being characterized and used in multiple studies,^18^^,^^37^^,^^38^ have not been used to examine developmental stages prior to MN specification, we included three control lines (BJ siPSD “BJ,” 1016A, and CRTD1) to establish a healthy baseline. Additionally, the BJ line used in numerous reports^18^^,^^35^^,^^37^^,^^38^^,^^39^^,^^41^^,^^42^^,^^43^^,^^44^^,^^45^ was genetically edited with a similar approach (Figure 1B).

### The C-terminus Clover reporter does not affect the main properties of SMN protein

To determine if the Clover reporter tag affects the biology of SMN protein, various quality control assays were conducted. SMN localizes in both the cytoplasm and nuclear foci called gems, which are associated with Cajal bodies,^46^ where it plays a key chaperone role in the biogenesis of snRNPs. Gems are composed of SMN proteins, gemins2–7, and snRNPs, which are involved in RNA transcription and processing.^8^ We first examined whether the Clover tag influenced SMN recruitment to nuclear gems or their formation. The BJ parental line and two BJ SMN-edited clones (#16 and #23) were differentiated into spinal MNs using an embryoid-body (EB)-based protocol.^37^^,^^47^ MN cultures were fixed and immunostained against SMN 4 and 8 days after plating. Automated imaging and quantification^37^ of thousands of MNs showed no change in the number of SMN+ gems per ISL1+ MN with the Clover tag at the earliest time point, but significantly more gems were detected in BJ SMN:Clover MNs after 8 days (Figures 1G–1I). This increase may be due to the higher fluorescence of SMN:Clover + gems compared to the antibody-labeled ones and therefore more easily identified by the image analysis script (Figures S2A and S2B). Importantly, no SMN protein aggregation was detected in hiPSCs or derived MNs. To investigate if the Clover tag affected gem dynamics, MNs from BJ SMN:Clover lines were treated with MLN4924 (which prevents SMN degradation^37^) or cycloheximide (CHX, which halts protein synthesis) and immunostained against SMN. MLN4924 increased the number of Clover+ gems, while CHX had the opposite effect (Figures S2C and S2D), indicating that Clover does not impact gem dynamics. Co-immunoprecipitation using anti-SMN or anti-GFP antibodies from BJ parental and edited BJ hiPSC lysates showed that SMN:Clover binds to untagged SMN, and gemin2, similar to untagged SMN (Figure 1J), does not interfere with SMN self-oligomerization and binding with major SMN complex components.

Next, we measured SMN protein half-life by treating parental SMA hiPSC lines and corrected clones with CHX, a method previously used to study SMN turnover^48^^,^^49^^,^^50^ that yielded ∼48 h in hiPSC-derived MNs.^37^ Automated imaging and quantification showed a half-life of ∼40 h for SMN:Clover in the corrected clones (Figures S2E–S2H), indicating that the fluorescent tag does not significantly alter SMN turnover. Similar treatment and quantification of immunostained SMN showed a comparable degradation profile for total SMN (untagged and tagged with Clover) with a ∼40 h half-life in BJ and 39C-III lines and isogenic clones (Figures S2I–S2K). However, the 50% decline in SMN protein levels for the 51N-II and 38D-I parental lines was reached later (Figures 1K–1M and S2L–S1M), possibly due to specific pathways regulating SMN protein when levels are below a certain threshold. Comparable cell number decline upon CHX treatment across lines indicated similar accumulated toxicity (Figures S2N–S2Q). Analogous degradation profiles were observed for gemin2 protein levels, showing a ∼45–50 h half-life in BJ WT hiPSCs and corrected clones, paralleling SMA parental lines (Figures S2R–S2Y). Furthermore, immunostaining-based analysis revealed a 3- to 4-fold increase in SMN protein in corrected hiPSCs compared to their parental lines (Figures 1K–1M and S2J), and tagging SMN with Clover did not alter Gemin2 protein levels in the corrected clones compared to parental lines (Figures S2R–S2Y). To validate SMN:Clover functionality, we measured snRNA levels involved in SMN-dependent splicing. There are two classes of spliceosomal snRNPs: the major spliceosome (U2 dependent) composed of U1, U2, U4/U6, and U5 snRNPs and the minor spliceosome (U12 dependent) formed by U11, U12, U4atac/U6atac, and U5 snRNPs.^51^ SMN deficiency preferentially reduces minor snRNPs levels,^52^^,^^53^ contributing to MN dysfunction in SMA.^54^ As in SMA mice this reduction is more pronounced in MNs^55^ and requires severe SMN decrease,^53^^,^^54^ we used MNs derived from the SMA type I line. While MNs derived from 3 healthy control hiPSCs showed similar RNA expression levels (Figure S3), SMA type I MNs showed a marked decrease primarily in minor snRNAs compared to healthy MNs, except for U11 (Figure 1N). This defect was corrected in the isogenic control MNs. In summary, we have not detected any abnormality in the corrected hiPSCs. Overall, these results indicate that endogenously tagged SMN:Clover displays subcellular localization, binding to key partners, turnover rate, response to modulators of its stability, and function analogous to untagged SMN.

### The corrected isogenic hiPSC lines present similar proliferation rates and differentiation potential to healthy control and SMA parental lines

Several studies have reported alterations in cell cycle genes in SMA models.^55^^,^^56^ Other publications have shown conflicting results on reduced^57^^,^^58^^,^^59^^,^^60^ or increased^61^^,^^62^ cell proliferation upon SMN loss depending on the cell type and the percentage of SMN reduction. We investigated whether the isogenic corrected SMA lines, with significantly higher SMN protein levels than the parental ones, displayed different growth profiles that could influence their differentiation capabilities. hiPSC colonies from the BJ control and the three SMA isogenic trios were dissociated and even single-cell suspensions were plated. Two days after plating, SiR-DNA, a cell-permeable fluorogenic probe for live-cell DNA labeling, was added and the cells were imaged. The same fields and wells were imaged 2, 4, and 6 days later, and the area occupied by the labeled hiPSC nuclei was automatically quantified. No significant difference was observed in the hiPSC colony growth rate between the disease and the BJ control lines (Figures S4A, S4E, S5A, and S4B), although the type I line exhibited increased vulnerability compared to the other lines, shown by the reduced number of cells that initially attached to the plate (Figure S4E). No significant difference was observed between the corrected clones and their isogenic SMA counterparts (Figures S4B–S4E and S5A). Quantification of P-H3, a commonly used mitotic marker, on fixed and immunostained plates at multiple time points after plating also revealed no robust difference between the SMA parental and the corrected clones (data not shown). Therefore, no morphological or growth abnormalities were detected in the SMA parental hiPSCs compared to BJ WT or between the isogenic corrected hiPSCs and the parental SMA ones. These data align with the argument that SMN’s control of cell cycle progression and cell proliferation is highly cell type specific.

Different hiPSC lines have varied predispositions to differentiate into the three germ lineages. Next, we ought to rule out that the genome editing introduced an undetected off-target effect, which could potentially alter the differentiation capabilities of the lines. The differentiation capacity of both the BJ WT and the three SMA lines has been separately published before.^37^^,^^38^ To do a side-by-side comparison, the same number of hiPSCs from the four lines was plated and cultured in STEMdiff Trilineage Differentiation media. To assess the hiPSC differentiation potential, we first validated that the expression levels of *SOX1* and *PAX6* (ectodermal markers); *TBXT*, *NCAM*, and *CXCR4* (mesodermal markers); and *SOX17* and *LEFTY1* (endodermal markers) genes were highest in the hiPSCs cultured with the corresponding differentiation medium (Figures S5C–S5I). Next, while significant differences were occasionally detected between the control line and one or two SMA lines, no consistent bias for any of the SMA lines toward a specific lineage was detected under these culture conditions (Figures S5J–S5P). Similarly, we did not detect robust differences in the expression levels of the lineage-specific genes measured between the corrected clones and their isogenic parental SMA lines (Figures S4F–S4H). Together, these results demonstrate that the isogenic corrected hiPSCs do not exhibit major differences in proliferation or three-germ layer differentiation potential compared to the parental lines.

### The progressive death of postmitotic SMA MNs is rescued in the corrected isogenic lines

**Figure 2:**
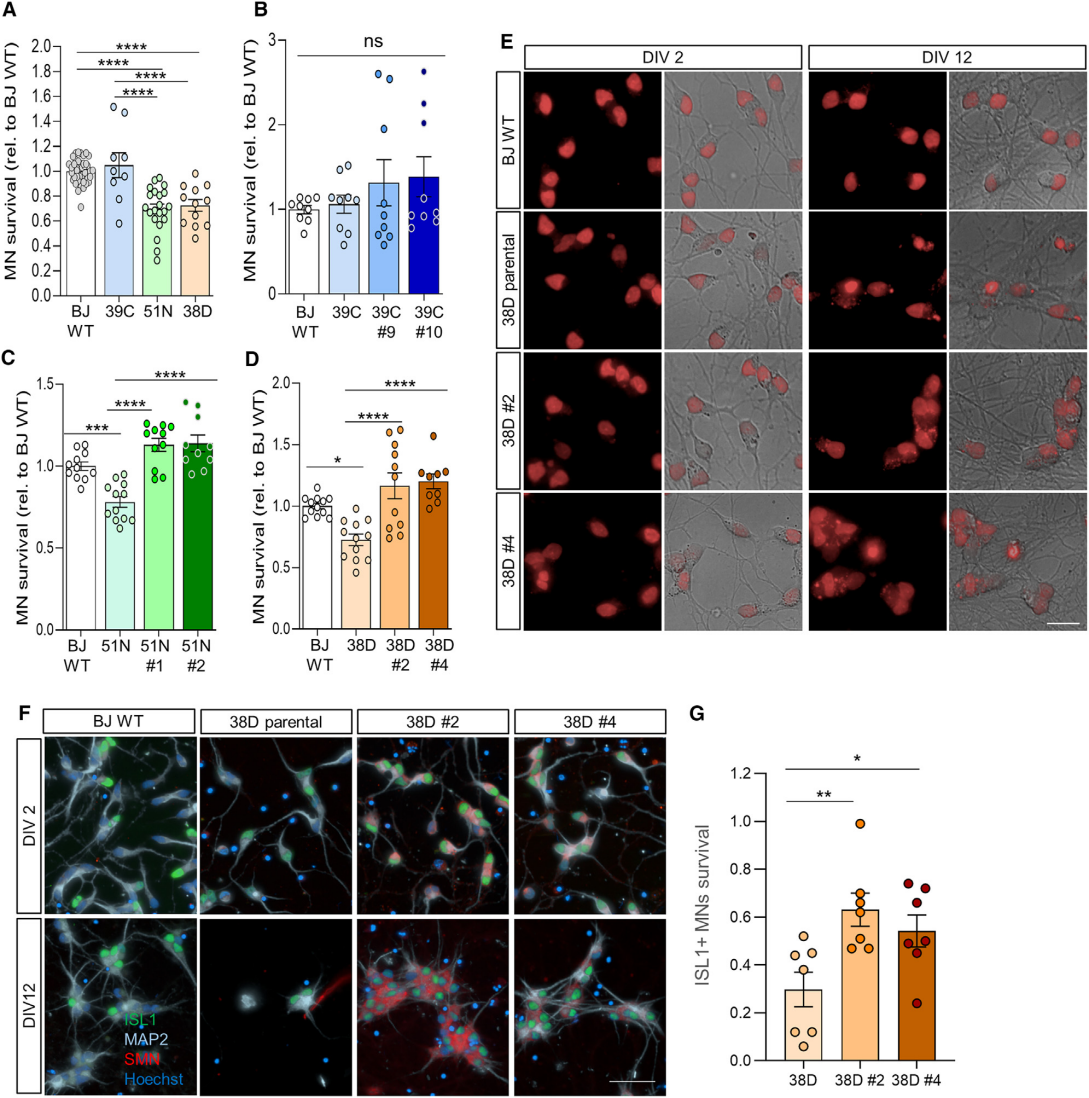
SMA hiPSC-derived spinal MN death is rescued in the isogenic corrected lines (A–D) Quantification of SiR-DNA-stained SMA hiPSC-derived MNs that survive 10 days after plating (A), 39C-III parental and its isogenic corrected MNs (B), 51N-II parental and its isogenic corrected MNs (C), and 38D-I parental and its isogenic corrected MNs (D). Each dot represents individually analyzed wells of all conducted experiments (*N* = 3–7, *n* = 3 wells per line per experiment. One-way ANOVA/Tukey’s analysis). (E) Representative SiR-DNA (red) stained MN cultures 2 and 12 days after plating. Scale bar, 20 μm. See also Figures S6A–S6F. (F) Representative immunostained MNs after 2 and 12 days in culture (ISL1, green; MAP2, cyan; SMN, red; nuclei blue). Scale bar, 50 μm. (G) Fraction of SMA 38D-I and isogenic ISL1+ MNs that survived after 12 days relative to the number quantified after 2 days in culture (one-way ANOVA/Fisher’s LSD analysis, *N* = 7). See also Figures S6G–S6H.

Although multisystemic abnormalities have been widely reported for SMA in the last decade,^63^^,^^64^ MNs are the most affected cell type by SMN deficiency, and their selective vulnerability is still under investigation.^65^^,^^66^^,^^67^ Previous studies^35^^,^^68^^,^^69^ and our own work^38^ have shown that SMA hiPSC-derived spinal MNs die faster in culture than those without *SMN1* mutations and that increasing SMN levels promotes MN survival.^37^ However, these assays were not performed in an isogenic context. To investigate MN death in our isogenic SMA model, we subjected the control line and three isogenic trios to an EB-based MN differentiation protocol previously published by us^18^^,^^37^ (Figure S6A), based on a foundational protocol for pluripotent cell differentiation into MNs.^47^ At the end of differentiation, spinal cord spheres were dissociated and the neurons were plated. To avoid variability in cell number quantification across plates, the same MN cultures were stained with SiR-DNA 2 days after plating and imaged the same day and 10 days later (12 days *in vitro*). Survival rates were calculated relative to initial plating numbers, showing that only 70% of 51N-II and 38D-I SMA MNs survived compared to the healthy control line (Figure 2A). The 39C-III SMA MNs did not show a significant reduction in survival compared to BJ WT (Figures 2A and 2B). Survival improvement in isogenic lines correlated with phenotype severity: 39C-III and 51N-II isogenic MNs showed a 20%–30% survival increase, while 38D-I lines showed a 50%–70% increase (Figures 2D, 2E, and S6B–S6F). This supports a greater potential rescue in more severe phenotypes. Importantly, compared to BJ WT, the corrected SMA MNs showed a complete rescue in survival (Figures 2C and 2D), demonstrating the functionality of the edited SMN protein. To confirm that SiR-DNA live-imaged cells were neurons, spinal cord spheres from the type I isogenic trio were fixed and immunostained after 2 and 12 days in culture. Over 95% of cells were MAP2+ neurons and ∼60% expressed ISL1 (Figures S6G–S6H). Using this approach, only ∼30% of SMA 38D-I MNs survived 12 days in culture, while isogenic clones showed ∼60% survival (Figures 2F and 2G). These results indicate that converting *SMN2* into *SMN1*, significantly increasing SMN protein markedly, ameliorates postmitotic MN death. While SMN deficiency is a major factor in MN death in SMA, other factors may also contribute. Thus, isogenic models offer a better approach to generate disease-relevant knowledge than using hiPSC lines from healthy donors for comparison.

### SMA hiPSCs show an impaired SCO formation, and the corrected isogenic lines partially restore that phenotype

**Figure 3:**
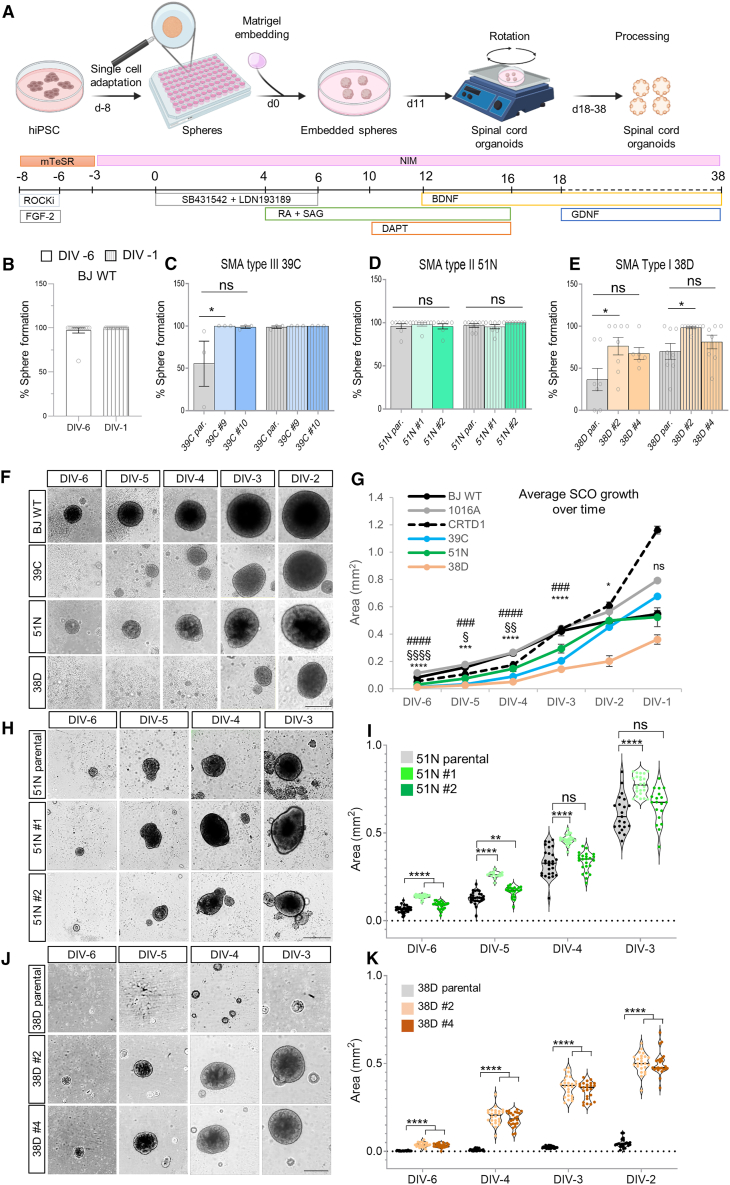
SMA hiPSCs present a defective sphere formation and growth when subjected to a spinal cord organoid protocol, which is improved in the isogenic corrected lines (A–E) (A) Schematic representation of the protocol followed to generate ventral spinal cord organoids. Percentage of hiPSC-seeded wells that formed a sphere two days after plating and after 7 days in culture for control BJ WT line (B), SMA 39C-III and isogenic corrected clones (C), 51N-II and corrected clones (D), and 38D-I and corrected clones (E) (*N* = 3–13). See also Figures S7A–S7D. (F) Representative self-assembled BJ WT and SMA spheres at the indicated developmental times. Scale bar, 400 μm. (G) Quantification of WT and SMA sphere area (mm^2^) over time. The graph represents the average of at least 30 spheres quantified per line and per experiment (# represents comparisons between BJ WT and 39C-III; § between BJ WT and 51N-II; and ∗ between BJ WT and 38D-I, *N* = 4–8). (H and I) (H) Representative SMA parental 51N-II and isogenic corrected self-assembled spheres at the indicated times (scale bar, 400 μm) and violin plot showing the size distribution of the individual spheres quantified for one representative experiment (I) (*n* = 30 spheres). (J and K) (J) Representative SMA parental 38D-I and isogenic corrected self-assembled spheres at the indicated times (scale bar, 400 μm) and sphere area quantification for one representative experiment (K) (*n* = 30 spheres). See also Figures S7E–S7L. Kruskal-Wallis analysis with Dunn’s test (B–E) and two-way ANOVA with Tukey’s multiple comparison test (G, I, and K) used for statistical analysis.

Once we demonstrated that our isogenic model recapitulates the most important SMA hallmark, MN death in a disease severity-dependent manner, and that *SMN2*-converted clones showed significant attenuation of this phenotype, we aimed to shed light on the unresolved question of how SMN protein is essential for the survival of MNs specifically. We adapted our EB-based differentiation protocol to generate a more robust model by reducing and controlling the starting number of hiPSCs, producing individually generated stem cell aggregates embedded in an extracellular matrix, maintaining the EB protocol’s patterning (Figure 3A). Using this ventral SCO (vSCO) protocol, we studied MN specification, maturation, and survival with our isogenic SMA lines, adding two healthy control lines (1016A and CRTD1) to better set a healthy baseline. SMN protein levels in these controls were analogous to BJ WT (Figures S7A and S7B).

We measured vSCO formation efficiency and growth over time as a proxy for organoid health. Organoids were imaged to determine the percentage of stem-cell spheres self-assembling 2 days after hiPSC seeding and after a week in culture. hiPSCs robustly self-assembled into detectable aggregates within 16 h (Figure S7C). WT hiPSCs showed 100% sphere formation (∼150 μm in diameter) (Figures 3B–3F and S7D), while only 40% of the severe SMA line 38D-I formed smaller, irregular shapes, increasing to 70% by the end of the assay (Figures 3E and 3F). The least severe line, 39C-III, showed ∼60% sphere formation initially, reaching 100% by completion (Figures 3C and 3F). The SMA line 51N-II, despite having the same *SMN2* copy number and similar SMN levels as type III (Figures 1D and 1E^37^), did not show a defect, although the spheres were initially smaller than the WT (Figures 3D and 3H). Corrected type I clones partially fixed the self-assembly deficiency, with 60%–80% initial formation and 80%–100% by completion for clones #4 and #2, respectively (Figures 3E and 3J). Corrected 39C clones showed full formation efficiency from the start (Figure 3C). The quantification of sphere growths over time confirmed that WT spheres were the largest from the beginning, 38D-I the smallest, and 51N-II displayed the mildest phenotype (Figures 3F and 3G). Isogenic corrected spheres were significantly larger than parental ones (Figures S7E and 4J–4K), with milder differences in 51N-II (Figures 3H and 3I). Despite two of the SMA hiPSCs showed robust self-assembly defects, spheres that survived grew similarly or faster than the WT (Figures S7F–S7I), suggesting that SMA hiPSCs do not have a general proliferation failure but an impaired self-assembly/survival capability.

To validate this, increasing numbers of hiPSCs were seeded and imaged 5 and 15 days later. While 600 hiPSCs were sufficient for BJ WT to form a uniform sphere, 38D-I required up to 5,000 cells (Figures S7J–S7K). The 39C-III line self-assembled less robustly, and 51N-II formed healthy-looking spheres at 600 cells, though smaller than BJ WT (Figures S7J–S7K). This early aggregation phenotype was also observed when subjecting the hiPSC to our EB protocol (Figure S7L). These results indicate that SMA hiPSCs, especially type I, have a deficient capacity to self-assemble into aggregates at low cell concentrations, hinting at severe defects in early embryonic development (as cell-to-cell adhesion forces are vital for morphogenesis of the embryo) when both *SMN1* alleles are mutated or deleted.

### SMA vSCOs show altered neurodevelopment, which is partially corrected in the isogenic control lines

While there has been controversy on whether SMA motor axons show defects in formation and outgrowth during development,^21^^,^^70^^,^^71^^,^^72^^,^^73^ recent studies described abnormalities in MN progenitors resulting in defective MN migration, target innervation, and survival.^22^^,^^23^^,^^74^ Neurogenesis defects in several brain areas have also been reported in SMA models.^58^ These findings, along with our observed impairments in SMA hiPSC self-organization into stem cell aggregates and reduced MN survival, led us to investigate how SMN deficiency impacts neural progenitors, neurogenesis, and MN differentiation in our isogenic human system.

**Figure 4:**
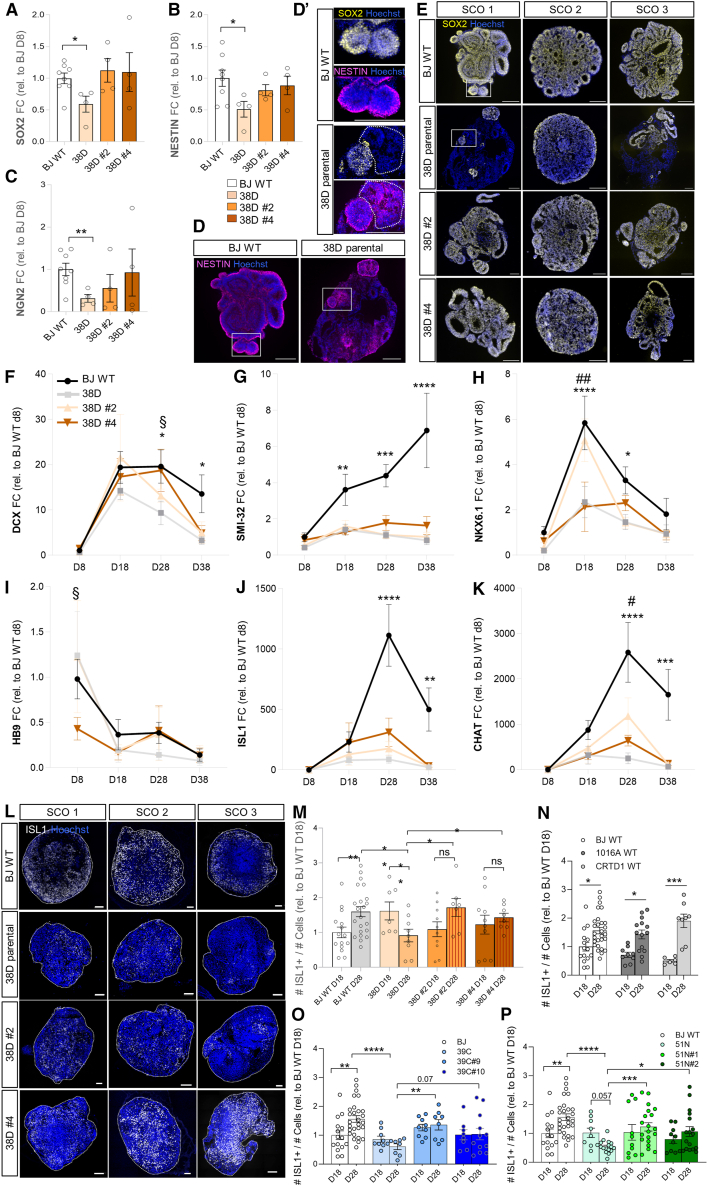
SMA spinal cord organoids show signs of altered neural development, a phenotype that is partially corrected in the isogenic controls (A–C) mRNA expression qPCR quantification of *SOX2* (A), *NESTIN* (B), and *NGN2* (C) in SCOs derived from BJ WT, SMA 38D-I, and isogenic corrected clones 8 days into the differentiation protocol (D8). Gene expression is indicated as fold change of 2-ΔΔCt with respect to 18 s, relative to BJ WT (*N* = 4, *n* = 4–8 pooled SCOs per experiment). (D) Representative D8 SCOs immunostained against NESTIN (magenta; nuclei stained with Hoechst, blue). Scale bar, 100 μm. White squares represent magnified areas displayed in (D′). Dotted line indicates a NESTIN+; SOX2− (yellow) SCO region. Scale bar, 100 μm. (E–K) (E) Representative SOX2 (yellow) immunostained D8 SCOs. Nuclei stained with Hoechst (blue). Scale bar, 100 μm. See also Figure S8 qPCR quantification of *DCX* (F), *SMI-32* (G), *NKX6.1* (H), *HB9* (I), *ISL1* (J), and *CHAT* (K) mRNA expression in SCOs derived from BJ WT, SMA 38D-I, and both isogenic corrected clones 8, 18, 28, and 38 days into the differentiation protocol (*N* = 4, *n* = 4–8 pooled SCOs per experiment. ∗ represents difference between BJ WT and 38D-I; # between 38D and 38D clone #2; and § between 38D and clone #4). (L–P) (L) Representative ISL1 (white) immunostained D28 SCOs (scale bar, 100 μm) and quantification of ISL1+ cells relative to total number of cells (Hoechst+) from D18-D28 38D-I isogenic trio vs. BJ WT (M), three healthy control lines (N), 39C-III isogenic trio (O), and 51N-II isogenic trio (P) vs. BJ WT (*N* = 3–6, *n* = 3–4 SCOs per experiment). See also Figure S9. One-way (A–C) or two-way (F–K and M–P) ANOVA with Fischer’s LSD multiple comparison test used for statistical analysis.

vSCOs from three healthy WT lines, SMA, and their isogenic corrected lines were generated following the previously described protocol (Figure 3A), and key gene regulatory networks governing MN specification were characterized longitudinally. No significant differences in mRNA levels of the NSC markers *SOX2* and *NESTIN* were observed between the control lines (Figures S8A–S8C), and immunostaining of vSCO cryosections showed no disparities either (Figures S8D and S8E). Expectedly, given that severe differences in organoid assembly were observed primarily in the severe SMA type I line, no major differences were detected between SMA 39C-III or 51N-II and BJ WT or their isogenic controls for these NSC markers (Figures S8F–S8J and S8K–S8O), which were, however, significantly reduced in the SMA 38D-I vSCOs (Figures 4A and 4B). Accordingly, *NGN2* mRNA levels, a pro-neural gene positively regulated by *SOX2*,^75^ were significantly downregulated (Figure 4C). The isogenic corrected clones partially corrected these phenotypes (Figures 4A–4C). Immunostaining confirmed these results and uncovered NESTIN+ cells negative for SOX2 in the SMA type I organoids (Figures 4D, 4E, and S8P). As the expression of both NSC genes during CNS development is commonly found concomitant,^76^ this might suggest that specific NSC subpopulations are altered upon severe SMN deficiency, potentially resulting in lineage specification abnormalities later in development.

We next explored whether the expression of pan-neuronal and MN-specific genes was altered throughout the development of the disease vSCOs. Using vSCOs derived from the 3 WT lines as reference, we observed expected changes in gene expression according to known genetic programs governing spinal cord development *in vivo*.^77^ *DCX*, an immature neuronal marker, increased from day 8 to day 18 and declined after day 28 (Figure S9A). *SMI-32*, a pan-neuronal marker, gradually increased during differentiation (Figure S9B). *NKX6.1*, a marker for spinal cord MN and ventral V3 progenitor domains, peaked at day 18 and decreased later (Figure S9C). *HB9*, a terminal MN transcription factor (TF),^78^^,^^79^ was highest at day 8 and decreased after that (Figure S9D). *ISL1*, another canonical MN identity TF and *CHAT*, marker for mature cholinergic MNs, increased from day 8, peaking at day 28 (Figures S9E and S9F).

Comparison of SMA to healthy vSCOs revealed reduced *DCX* expression at all time points for the three disease types (Figures 4F, S9A, S9G, and S9M). Similar reductions were observed for *SMI-32* (Figures 4G, S9B, S9H, and S9N). *DCX* expression was notably restored in the respective isogenic vSCOs; however, *SMI-32* levels in the isogenic controls remained comparable to the parental disease, especially for type I. *NKX6.1* expression was significantly reduced compared to isogenic controls and WT, especially in type I (Figures 4H, S9C, S9I, and S9O). *HB9* was higher in type II and III vSCOs (Figures S9D, S9J, and S9P) and showed an upward trend in type I at the earliest time point studied (Figure 4I). *ISL1* and *CHAT* were significantly decreased in all SMA vSCOs compared to healthy controls (Figures 4J, 4K, S9E, S9F, S9K, S9L, S9Q, and S9R) and peaked at day 18 in SMA vSCOs instead of day 28, which aligns with an earlier *HB9* expression and points to accelerated MN differentiation in the disease vSCOs. The isogenic corrected clones displayed intermediate profiles between healthy and SMA vSCOs for these 4 progenitor and MN markers. Immunostaining confirmed reduced ISL1+ cells in day 28 SMA vSCOs compared to the healthy WTs and corrected vSCOs (Figures 4L–4P and S9S–S9U). Fitting the transcription analysis, ISL1+ MN numbers per vSCO were higher at day 18 than at day 28 in types I and II, whereas the opposite was observed in WT and isogenic corrected vSCOs (Figures 4L–4N and 4P), reinforcing an abnormal MN specification timeline in SMA. Reductions in NKX6.1+ (Figures S9V and S9W) and MAP2+ (Figure S9X) cells were also observed in SMA vSCOs, with phenotype amelioration in isogenic controls.

Together, these results show defective MN specification programs in SMA. Further, the isogenic control vSCOs generally follow the expected non-diseased developmental patterns. However, some disease phenotypes are not permanently corrected despite having significantly higher SMN amounts than their diseased counterparts. It is crucial to note that while several healthy control lines, used in numerous studies,^18^^,^^35^^,^^37^^,^^38^^,^^41^^,^^42^^,^^43^^,^^80^^,^^81^ were employed to define a normal developmental baseline, they have different genetic backgrounds from the SMA lines. Therefore, the most relevant biological comparisons are those made between each SMA line and its isogenic corrected controls.

### Longitudinal scRNA-seq reveals a mesodermal specification bias in SMA neuromuscular SCOs at the expense of neural lineages

**Figure 5:**
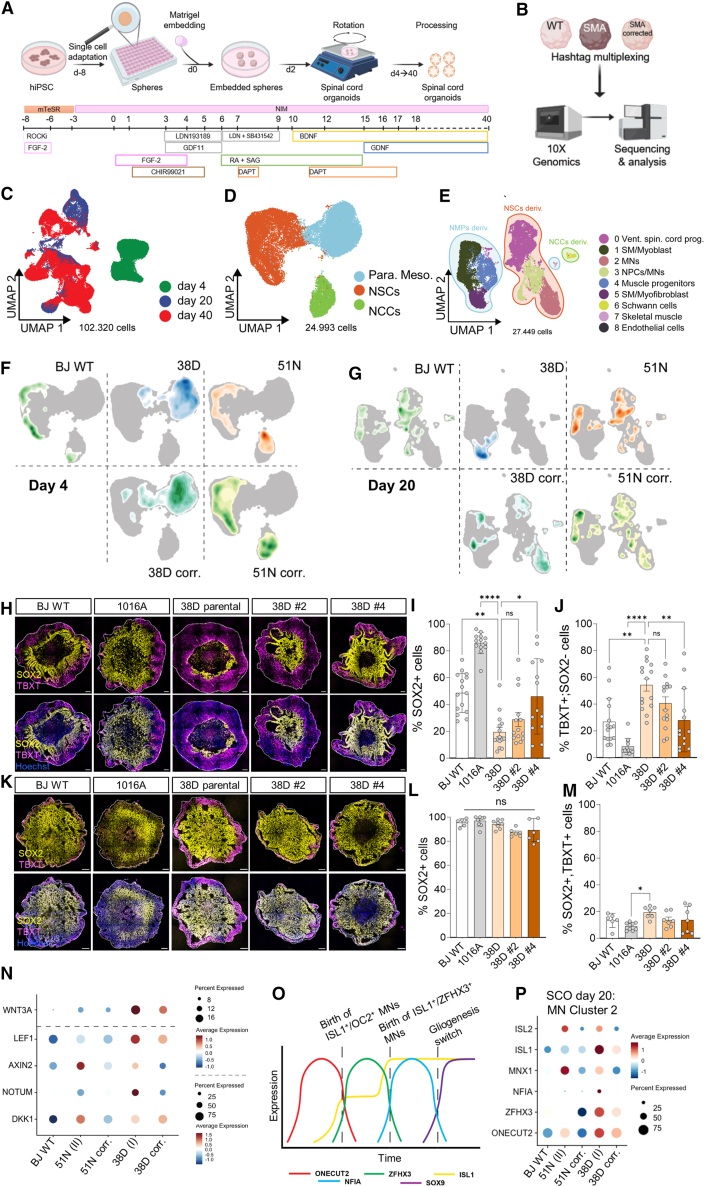
Longitudinal single-cell transcriptomic analysis reveals an NMP misspecification in SMA type I SCOs favoring a mesodermal lineage commitment (A) Schematic representation of the protocol followed for the generation of NMP-derived SCOs. (B) Schematic workflow of the single-cell transcriptomic analysis. (C–H) (C) Uniform manifold approximation and projection (UMAP) dimensionality reduction of the entire dataset (all control and disease samples at three developmental time points). UMAP representation of sequencing data obtained from all organoids at days 4 (D) and 20 (E). Density plot overlaid on the UMAP embedding of the main cell types at days 4 (F) and 20 (G) for BJ WT, SMA 38D-I, SMA 51N-II, and their combined two isogenic control SCOs. (H) Representative SOX2 (yellow) and TBXT (magenta) immunostained day 4 SCOs. Nuclei stained with Hoechst (blue). Scale bar, 100 μm. (I and J) (I) Quantification of the percentage of SOX2+ cells (NSCs and NMPs) and (J) TBXT+; SOX2− cells (NMPs committed to mesodermal lineage) (*N* = 3–4, 3–4 SCOs per experiment). (K–M) (K) Representative SOX2/TBXT immunostained day 2 SCOs and quantification of the percentage of SOX2+ cells (L) and SOX2+; TBXT+ double-positive cells (NMPs) (M) (*N* = 3–4, 3–4 SCOs per experiment). (N) Dot plot showing expression levels of key genes of the canonical WNT pathway from all cells of day 4 SCOs. (O) Schematic representation of the temporal expression of key transcription factors in the neural patterning of the spinal cord. (P) Dot plot showing expression levels of key genes for MN specification in “MN cluster 2” of day 20 SCOs. See also Figures S10 and S11. One-way ANOVA with Fischer’s LSD multiple comparison test used for statistical analysis.

Having observed significant abnormalities in NSCs, MN progenitors (pMNs), and MNs in SMA vSCOs, we next explored how SMN deficiency influenced neural lineage specification. To increase the cellular complexity of our vSCOs and therefore enhance the probability of identification of affected progenitor populations, we developed a more complex SCO model. This involved inducing NMPs^82^^,^^83^^,^^84^ from hiPSCs before patterning them into the spinal cord (Figure 5A and STAR Methods). NMPs, or axial stem cells, are bipotent progenitors, localized in the tail bud region of the developing embryo and are essential for axial elongation, giving rise to neural and mesodermal lineages^85^^,^^86^ and neural crest cells that can generate Schwann cells,^87^ essential for the maturation and maintenance of neuromuscular junctions.^88^ To determine the major cell populations at different stages, day 4, 20, and 40 neuromuscular SCOs from BJ WT, 51N-II, 38D-I, and their isogenic corrected clones were subjected to scRNA-seq analysis (Figure 5B). In total, 102,320 cells were analyzed (Figure 5C). On average, 16,170 unique transcripts were determined, representing 4,799 genes per cell. We identified 3 main cell clusters on day 4 (Figures 5D and S10A, S10B) and 9 at day 20 (Figures 5E, S10C, and S10D). The three main cell clusters at day 4 corresponded to paraxial mesodermal progenitors (cluster 0), neuroectodermal cells (NSCs) (cluster 1), and neural crest cells (NCCs) (cluster 2) (Figure S10A). Interestingly, while most cells of the control SCOs were committed to a neuroectodermal or NCC lineage by day 4, SMA 38D-I SCOs showed enrichment in the mesodermal cluster and a decrease in the other two (Figures 5D–5F and S10B). SCOs from the mild SMA line were more similar to healthy controls than to severe SMA SCOs (Figures 5D–5F and S10B), and the 38D-I isogenic corrected SCOs partially corrected the disease mesodermal bias.

Cell type frequencies in heatmaps showed that while 83.85% of BJ WT SCOs were committed to an NSC identity and ∼0.6% were paraxial mesoderm progenitors, the 38D-I SCOs had 12.58% and 87.30%, respectively, and the corrected clones had 22.85% and 77.10% (Figure S10B). This mesodermal lineage bias in 38D-I SCOs led to severe cell identity differences as the organoids developed. Immunostaining confirmed that while >50% of cells were SOX2+ in healthy controls and ∼25% were committed to a mesodermal fate (TBXT+; SOX2−), in SMA 38D-I, only 20% were SOX2+ and almost 60% were mesodermal (Figures 5H–5J). This was reversed in the isogenic corrected clones, especially clone #4. To rule out that the WT hiPSCs bypassed the NMP stage, day 2 SCOs were also analyzed, showing >90% pluripotent SOX2+ cells, of which 20% had an NMP identity (SOX2+; TBXT+) (Figures 5K–5M) without significant differences between healthy and diseased SCOs being observed. This indicates that all hiPSC lines responded to WNT/FGF2 induction similarly. The WNT signaling pathway plays a pivotal role in guiding vertebrate axial development,^89^ both by specifying the fate of neural progenitors in posterior spinal cord segments and by governing the initiation and maintenance of mesodermal specification programs. Importantly, WNT signaling directs NMPs toward paraxial mesodermal fates.^85^^,^^90^ Given the neuromesodermal misspecification detected in SMA-I SCOs, we analyzed WNT pathway expression levels using our scRNA-seq data. WNT3A and WNT/β-catenin pathway downstream effectors were higher expressed in SMA SCOs, in a disease severity manner (Figure 5N), fitting the hypothesis of a dysregulated WNT activity underlying the neuromesodermal misspecification we have detected in SMA and in agreement with the accumulation of β-catenin in an SMA mouse model.^91^ Further studies are needed to understand the contribution of this critical signaling pathway in SMA’s early pathology.

By day 20, 38D-I SCOs showed a marked decrease in ventral spinal cord progenitors, MNs, and neural progenitor cells (NPCs) (clusters 0, 2, and 3, respectively) (Figures 5E–5G, S10C, and S10D), with increased muscle progenitor and smooth muscle cells (clusters 4 and 5) (Figures 5E–5G, S10C, and S10D). Isogenic control SCOs showed amelioration of the disease phenotype, shifting neural lineage clusters over mesodermal ones, though not fully matching the healthy control (Figures S10B and S10D). Interestingly, SMA SCOs showed increased early, mid, and late-born spinal cord neuron markers^77^^,^^92^ within the MN cluster 2 (*ONECUT2*, *ZFHX3*, and *NFIA*) compared to controls (Figure 5O and 5P), suggesting earlier NPC differentiation into MNs in SMA, consistent with our vSCO model findings (Figures 4I–4N and S9D–S9F, S9J–S9L, S9P, and S9R). Similar patterns were observed for the MN markers *MNX1* (*HB9*) and *ISL1* (Figure 5P).

**Figure 6:**
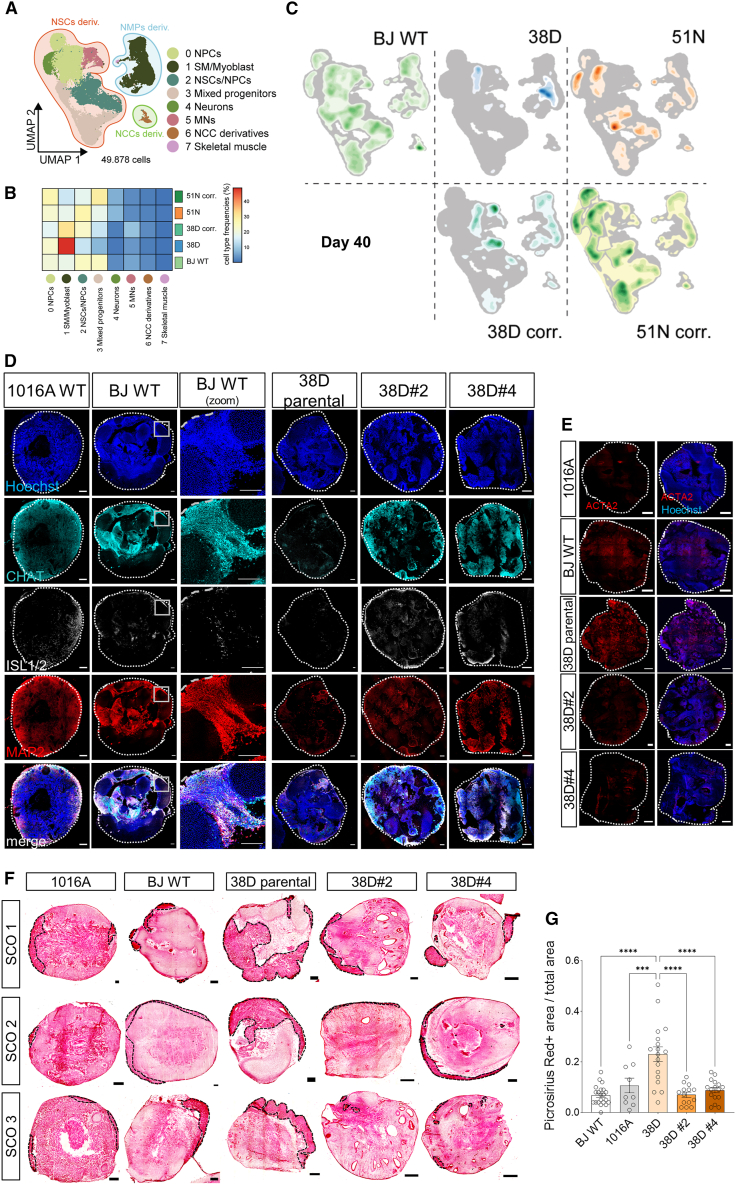
SMA NMP mesodermal bias in early SCOs results in a reduced number of MN/neural cell clusters in favor of muscle cells in the mature organoids (A) UMAP representation of sequencing data obtained from control and disease organoids at day 40 of the differentiation protocol. (B) Heatmap of cell frequencies of each cluster for day 40 SCOs. (C) Density plot overlaid on the UMAP embedding of the main cell types in day 40 SCOs. (D) Representative immunostained day 40 SCOs showing CHAT (cyan), ISL1 (white) (MN markers), and MAP2 (red) (neuronal marker). Nuclei stained with Hoechst (blue). Scale bar, 100 μm. Squared regions in the BJ WT panels are shown in magnified images. Scale bar, 100 μm (*N* = 3–4, 3–4 SCOs per experiment). (E) Representative immunostained day 40 SCOs showing myofibroblast marker ACTA2 (alpha-smooth muscle actin) (red). (F) Representative Picrosirius red collagen stained day 40 SCOs (dotted lines indicate Picrosirius red+ areas). Scale bar, 100 μm. (G) Quantification of the area positively stained over total SCO area (one-way ANOVA/Fisher’s LSD multiple comparison test, *N* = 4–5, 3–4 SCOs per experiment).

By day 40, we identified 8 main cell clusters (Figures 6A, 6B, and S10E). As organoid maturation proceeded, the mesoderm versus neural population bias in 38D-I SCOs remained (Figure 6C). Heatmaps showed that ∼45% of BJ WT SCO cells were NPCs, ∼9% were MNs, and ∼20% had a muscle identity. In 38D-I SCOs, ∼36% were NPCs, ∼4.5% were MNs, and 50% were muscle cells (Figures 6B and S10E). The corrected isogenic SCOs showed intermediate cell distribution, with 40.5% NPCs, ∼10% MNs, and ∼32% muscle cells (Figures 6B and S10E). Immunostaining of day 40 SCOs confirmed that all hiPSCs generated spinal MNs (ISL1+, CHAT+), neural (SMI-32+, MAP2+), and skeletal muscle cells (DESMIN+, TITIN+) (Figures 6D and S11). SMA type I SCOs showed reduced number of neuronal/MN cells compared to healthy controls, which was notably ameliorated in isogenic controls (Figures 6D and S11). While no drastic difference in DESMIN+ or TITIN+ cells was observed between SMA SCOs and controls, expectedly as only less than 2% of the day 40 SCO cells had a skeletal muscle identity (cell cluster 7 in Figures 6A, 6B, S10E, and S11), larger areas in SMA type I SCOs compared to controls were smooth muscle cells, indicated by ACTA2 and picrosirius red staining (Figures 6E and 6F–6G). The reduction in neural cell types suggests faulty neural specification in SMA SCOs, while the abnormal muscle cell predominance may indicate underlying defects in neuromuscular junction formation and maintenance^93^ as well as in mesodermal-derived tissues, aligning with SMA systemic alterations.^73^^,^^94^ Together, this longitudinal analysis has uncovered an early progenitor misspecification linked to severe SMA.

### The early neuromesodermal fate commitment defects observed in our SMA SCOs are recapitulated *in vivo*

**Figure 7:**
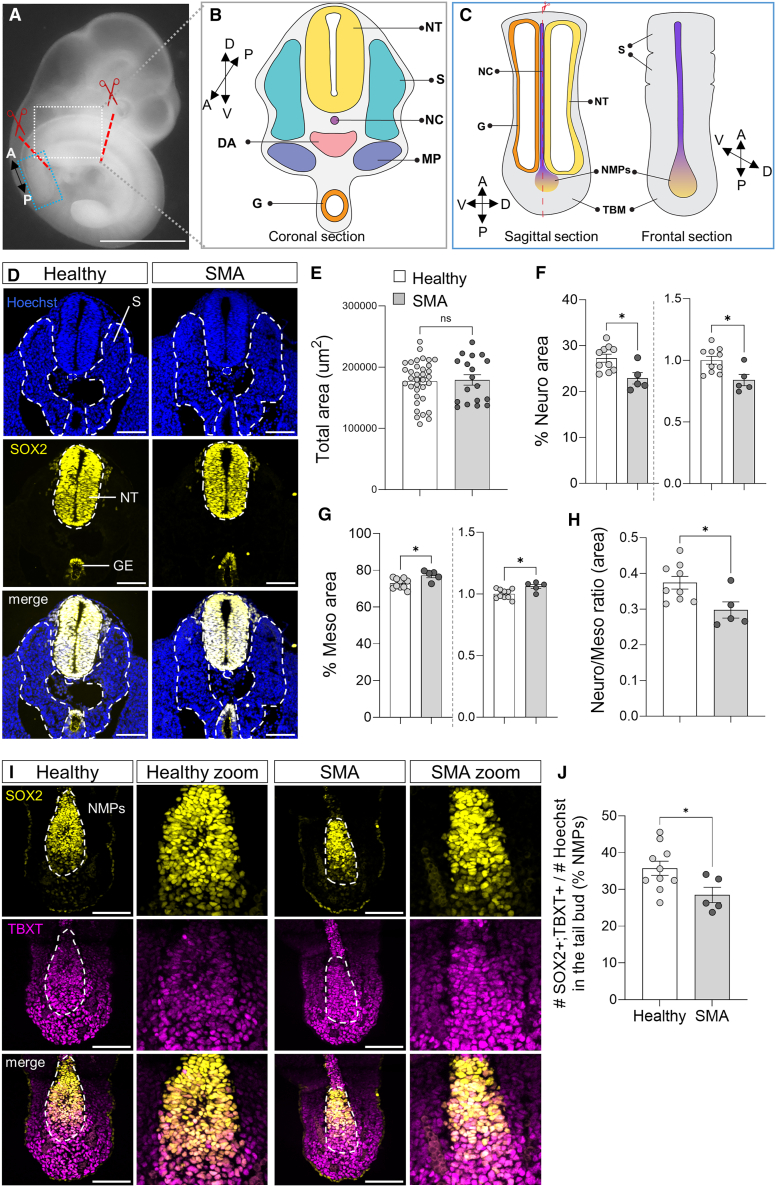
Early neuromesodermal fate commitment defects in SMNΔ7 mouse embryos (A–C) (A) Whole-mount image of an E10.5 mouse embryo. Scale bar, 1 mm. The white boxed area indicates the region sectioned coronally to determine neural and mesodermal regions, schematically represented in (B). The blue boxed area corresponds to the caudal progenitor zone, sectioned laterally to identify NMPs, schematically represented in (C). (D) Representative SOX2 (yellow) immunostained coronal sections of E10.5 spinal cord caudal segments. Nuclei stained with Hoechst. Scale bar, 100 μm. (E–H) (E) Quantification of the total spinal cord section (*N* = 10:5 heterozygote:SMA embryos, 4 quantified sections per embryo are shown). Quantification of the percentage of area occupied by neural tube (SOX2+) (left, relative to total spinal cord area; right, “SMA” relative to “Healthy”) (F) and by mesodermal tissue (SOX2−; Hoechst+) (G) in caudal spinal cord coronal sections and the ratio of neural vs. mesodermal regions (shown as in F) (H) (*N* = 10:5 heterozygote:SMA embryos). (I) Representative SOX2 (yellow), TBXT (magenta) immunostained lateral sections of E10.5 embryo tail bud regions. Nuclei stained with Hoechst. Scale bar, 100 μm. (J) Quantification of the percentage of NMPs (SOX2+; TBXT+) over the total number of cells (Hoechst+) localized at the tail bud (*N* = 10:5 heterozygote:SMA embryos). Unpaired two-tailed t test used for statistical analysis. A, anterior; D, dorsal; DA, dorsal aorta; G, gut; MP, mesonephros; NC, notochord; NT, neural tube; P, posterior; S, somite; TBM, tail bud mesoderm; V, ventral.

Next, we investigated whether the neuromesodermal specification defect observed in our isogenic model *in vitro* is recapitulated *in vivo*. During mouse embryo axial elongation, NMPs are found in the tail bud region between embryonic days E8.5 and E12.5, contributing cells to the elongating neural tube, presomitic mesoderm, and tail bud mesoderm^84^ (Figures 7A–7C). We used E10.5 SMNΔ7 embryos, the most common SMA mouse model,^95^ and heterozygote control littermates to study early spinal cord formation. We measured the neural tube area relative to the adjacent mesodermal tissue area. Coronal sections of the posterior end of the elongating spinal cord (somites 22–30) showed similar total areas between both genotypes (Figures 7D and 7E). However, SMA E10.5 embryos exhibited a significant 16% decrease in the neural tube size compared to healthy heterozygotes (Figures 7D–7F). This reduction was accompanied by a proportional increase in the size of neighboring somitic regions (mesodermal tissue) (Figures 7D and 7G), resulting in a reduced neural-to-mesodermal ratio (Figure 7H). These findings align with our *in vitro* results on neuromuscular organoids and again indicate aberrant NMP misspecification.

Finally, we quantified the abundance of NMPs in the embryos’ tail buds, finding a significant reduction in SMA embryos compared to healthy heterozygotes (Figures 7I and 7J). These results suggest that the NMP pool may be exhausted earlier in SMA embryos and that SMA NMPs display a preferential commitment toward a mesodermal lineage.

## Discussion

How the disturbance of a basic homeostatic function, such as mRNA splicing, leads primarily to a neuromuscular disorder remains puzzling. Efforts have been devoted to identify MN-specific functions of SMN to explain their selective vulnerability.^20^ However, there is still no consensus on whether this selectivity is solely due to cell-intrinsic abnormalities or whether cell non-autonomous mechanisms play a central role, let alone whether developmental marks render those neurons to degenerate months or years after having developed. We hypothesized that MN degeneration in SMA is imprinted during early development. We generated the first cohort of isogenic SMA hiPSC lines, used hiPSC-derived MN cultures, developed two SCO models, and performed longitudinal single-cell transcriptomics and found early developmental defects in SMA SCOs, characterized by derangements in neuronal cell specification. Additionally, we discovered abnormal neural/mesodermal tissue ratios in the neuromuscular organoids and in an early embryonic stage of a severe SMA mouse model, along with the decreased number of bipotent axial progenitors in the elongating spinal cord during embryonic development. These findings suggest altered maintenance and differentiation dynamics in the axial progenitor population. These insights constitute a foundation for future studies to uncover molecular events governing early developmental defects preceding SMA progression and selective MN loss. They also suggest that not all neurological alterations in patients with SMA might be resolved by increasing SMN protein postnatally. Surprisingly, *SMN2*-to-*SMN1* conversion in a well-characterized SMA type I line was not sufficient to fully revert the observed stem cell self-assembly and misspecification defects. This lack of full rescue fits with observations recently seen in an SMA mouse model where base editors were used to correct the *SMN2* C6-to-T nucleotide change,^96^ and, in our model, may be caused by additional mutations other than *SMN1* in the donors of the SMA lines contributing to SMA pathogenesis^97^^,^^98^ or by epigenetic changes caused by early SMN deficiency^99^^,^^100^^,^^101^^,^^102^^,^^103^ not erased during dedifferentiation protocols.

Previous studies have demonstrated organoid models’ potential for studying and treating human diseases.^104^^,^^105^ Worth noting, a multi-study proteomic profile showed greater overlap among human SMA samples than between human and mouse tissues,^106^ reinforcing the use of human-derived models for studying human disease. Isogenicity is crucial for causally validating gene roles and uncovering disease-relevant mechanisms. However, SMA’s complex genetics have hindered the development of this asset. Localized in an unstable region of chromosome 5q (11.1–13.3), *SMN* genes comprise 62% interspersed repetitive DNA, and the density of Alu elements is 4-fold higher than average in the genome, making this region particularly prone to rearrangements.^107^^,^^108^ We overcame this genetic challenge by using a newly generated cloning vector that enabled C-terminal tagging of the converted *SMN2*-to-*SMN1* gene with a fluorescent reporter, facilitating the live detection and quantification of full-length SMN protein and the identification of successfully edited cells. In addition to two isogenic, SMN-restored clones per parental SMA hiPSC line, we included several independent control lines to help us define a healthy phenotype. This toolkit allowed us to generate complex and robust patient-derived organoids apt to investigate the function of SMN at early developmental stages.

Neurodevelopmental abnormalities have been linked to late-onset neurodegenerative conditions.^109^^,^^110^ Studies in SMA animal models and human tissue show impaired perinatal development of cerebral regions^58^^,^^73^ and developmentally immature MN axons.^21^^,^^111^ Although three approved therapies for SMA^94^ can delay disease progression, patient responses to the treatment vary, and they can cause severe adverse effects without providing a definitive cure, especially for severe SMA.^94^^,^^112^^,^^113^ Non-responders might present extensive damage by the time therapy is applied, with higher MN degeneration priming during development. Understanding when and in which cell type the disease first manifests is crucial for optimizing therapies, potentially allowing *in utero* treatments for severe SMA, as shown for other genetic diseases.^114^

In our study, two of the three SMA lines displayed severe morphokinetic defects during stem cell aggregate self-assembly. These structures are likely more sensitive than *in vivo* organ development, with even mild defects in cell specification having dramatic consequences. Nevertheless, these abnormalities at an early developmental stage are consistent with dramatic phenotypes reported in severe SMA mouse models, in which 75% of mice died within 6 h of birth, with intra-utero death suggested.^115^^,^^116^ We also discovered disruptions in neuronal cell specification. SMA vSCOs presented abnormal NSC emergence, leading to neuronal progenitor deficiency and time-shifted expression of pMN and MN markers, which could indicate aberrant MN differentiation, resulting in quicker degeneration of postmitotic MNs, consistent with our MN survival assays. These results align with previous evidence showing altered protein expression profiles in SMA hiPSC-derived MN cultures.^106^ Longitudinal single-cell gene expression profiling, cell population distribution, and histological analysis revealed significant differences in SMA type I neuromesodermal SCOs compared to healthy controls and milder SMA forms from the earliest developmental phase, the NMP stage. NMPs in SMA-I SCOs showed a preferred commitment toward mesodermal specification and aversion to neural fates, leading to a muscle cell lineage bias as the SCOs developed. These results align with our findings in E10.5 SMNΔ7 mouse embryos, which showed a reduced NMP pool in the tail bud compared to healthy littermates. This, along with decreased neural tube size and enlarged surrounding mesodermal tissue, suggests that NMPs in severe SMA might get exhausted early and acquire an unbalanced mesodermal fate, a defect that had not been previously detected in SMA mice.

Our findings are coherent with and could explain the origin of immature MNs^21^ and abnormal myogenesis^117^^,^^118^ reported in SMA fetuses, as well as previous findings of pathology in multiple mesodermal-origin organs, including the musculoskeletal system, in patients with SMA.^119^ It would be interesting to determine if these pathological marks are selectively present in the vulnerable MN subpopulations in patients with SMA during development. Longitudinal single-cell multi-omics and lineage tracing might be crucial to elucidate the signaling pathways responsible for the altered early development in severe SMA and ultimately unravel the molecular mechanisms underlying selective MN vulnerability. We believe that our isogenic SMA organoid platform will enable dissecting SMN’s molecular function on MN development and survival; additionally, it could be leveraged for screening and molecular validation of new putative therapies aimed at ameliorating pathological developmental phenotypes.

### Limitations of the study

Our SCO model represents a significant advance in the SMA field, addressing limitations of current SMA *in vitro* systems. However, it lacks tissues like immune cells or vascular networks that may affect progenitor specification and differentiation. A similar single-cell transcriptomic analysis should be conducted on refined organoid or assembloid models. We generated isogenic lines from hiPSCs derived from patients with SMA type I, II, and III to cover the spectrum of disease severities, using in total 12 different lines (SMA, isogenic corrected, and healthy controls). Based on our findings, a similar analysis on the earliest spinal cord developmental phases should be done using multiple type I and isogenic corrected lines. Our assays testing SMN:Clover protein showed no functional deficits. However, to rule out any undetected protein abnormalities causing incomplete rescue of developmental defects, a similar study should be done with SMN:P2A-engineered hiPSCs. The neuromesodermal fate commitment defect in our SCO model, mirrored in a severe SMA mouse, aligns with studies on SMA human postmortem samples of later developmental stages^117^^,^^118^^,^^120^ and neonates,^21^ supporting a developmental origin for SMA hallmarks. To confirm clinical relevance, an analysis of early fetal development stages (CS11-15) is needed.

## STAR★Methods

### Key resources table

**Table:** 

REAGENT or RESOURCE	SOURCE	IDENTIFIER
**Antibodies**		

Mouse anti-IgG	Santa Cruz	sc2025; RRID: AB_737182
Mouse anti-SMN	Novus Biologicals	NB100-1936; RRID: AB_531374
Rabbit anti-Gemin2	Abcam	ab150383; RRID: AB_3099716
Rat anti-SOX2	Invitrogen	14-9811-82; RRID: AB_11219471
Mouse anti-NESTIN	Millipore	MAB353; RRID: AB_94911
Rabbit anti-ISL1	Abcam	ab109517; RRID: AB_10866454
Mouse anti-NKX6.1	Developmental Studies Hybridoma Bank	F55A10; RRID: AB_532378
Mouse anti-MAP2	Novus Biologicals	NBP2-25156; RRID: AB_3099715
Rabbit anti-TBXT	Cell Signaling	81694; RRID: AB_2799983
Mouse anti-ACTA2	Sigma-Aldrich	A2547; RRID: AB_476701
Rabbit anti-CHAT	Proteintech	20747-1-AP; RRID: AB_10898169
Mouse anti-SMI32/anti-Neurofilament H (NF-H)	Biolegend	801701; RRID: AB_2564642
Rabbit anti-Desmin	Abcam	ab15200; RRID: AB_301744
Mouse anti-Titin	Novus Biologicals	NB600-1206; RRID: AB_10003288
Mouse anti-β-Actin	Cell Signaling	8H10D10; RRID: AB_2242334
Rabbit anti-β-Tubulin	Cell Signaling	2146; RRID: AB_2210545

**Chemicals, peptides, and recombinant proteins**		

mTESR1	STEMCELL Technologies	85850
ReLeSR	STEMCELL Technologies	100–0484
Advanced DMEM/F12	Thermo Fisher Scientific	12634028
Neurobasal Medium	Thermo Fisher Scientific	21103049
GlutaMAX	Thermo Fisher Scientific	35050087
2-Mercaptoehtanol	Life Technologies	21985023
GlutaMAX	Thermo Fisher Scientific	35050087
Ascorbic Acid	SigmaAldrich	A4403
B27	Thermo Fisher Scientific	17504044
N2	Thermo Fisher Scientific	17502048
Penicillin-Streptomycin	Life Technologies	15140122
Matrigel	Corning	354234
TRIzol	Life Technologies	15596026
Laminin	Thermo Fisher Scientific	23017015
Poly-D-lysine	Sigma-Aldrich	A-003-E
TissueTek OCT	Sakura Finetek	4583
Sucrose	Millipore	107651
MLN-4924	Active Biochem	MLN-4924
Cycloheximide	Sigma-Aldrich	C1988
Y-27632 (ROCKi)	Hölzel	A11001-50
FGF-2	Millipore	32160702
SB431542	Bio-Techne	1614/10
LDN193189	Hölzel	M1873)
RA	Sigma-Aldrich	R2625
SAG	Millipore	566660
Ara-C	Sigma-Aldrich	C1768
DAPT	Tocris	2634/10
BDNF	Qkine	QK050
GDNF	Qkine	QK051
Papain	Worthington	LK003178
DNase	Worthington	LK003172
Accutase	Corning	25-058-Cl
Vectashield Antifade Mounting Medium	Biozol Diagnostica	VEC-H-1000
SiR-DNA	Spyrochrome	SC007
Hoechst33342	Life Technologies	H3570

**Critical commercial assays**		

STEMdiff™ Trilineage Differentiation Kit	STEMCELL Technologies	05230

**Deposited data**		

scRNA-seq	This paper	EGAS00001007259

**Experimental models: Cell lines**		

BJ siPS-D	Laboratory of Lee L. Rubin (Rodriguez-Muela et al.^18^^,^^37^)	HVRDi005-A, Table S2
1016A	Laboratory of Lee L. Rubin (Rodriguez-Muela et al.^18^^,^^37^)	HVRDi007-A, Table S2
38D-I	Laboratory of Lee L. Rubin (Rodriguez-Muela et al.^18^^,^^37^)	HVRDi015-A, Table S2
51N-II	Laboratory of Lee L. Rubin (Rodriguez-Muela et al.^18^^,^^37^)	HVRDi017-A, Table S2
39C-III	Laboratory of Lee L. Rubin (Rodriguez-Muela et al.^18^^,^^37^)	HVRDi016-A, Table S2
CRTD1	CRTD Stem Cell Core Facility (Volkner et al.^81^)	CRTDi004-A, Table S2
BJ-SMN:Clover	This paper	N/A
38D-I isogenic corrected clone #2	This paper	N/A
38D-I Isogenic corrected clone #4	This paper	N/A
51N-II Isogenic corrected clone #1	This paper	N/A
51N-II Isogenic corrected clone 2	This paper	N/A
39C-III Isogenic corrected clone 9	This paper	N/A
39C-III Isogenic corrected clone 10	This paper	N/A

**Experimental models: Organisms/strains**		

Mouse: Smn+/−;hSMN2+/+; hSMNΔ7+/+; Hb9:GFP+	Rodriguez-Muela et al.^18^^,^^37^	N/A

**Oligonucleotides**		

Single-stranded DNA oligos for cloning	IDT	See Table S3
Primers for PCR and Sanger Sequencing	IDT	See Table S3
Primer for RT-qPCR	IDT	See Table S3

**Recombinant DNA**		

pTG-Cr-SMN	This paper	N/A
pTG-HR-SMN:Clover	This paper	N/A

**Software and algorithms**		

GraphPad Prism 10.0.3	Graphpad Software, Inc.	https://www.graphpad.com/features
Geneious Prime 2023.0.3	Biomatters Ltd	https://www.geneious.com/
ImageJ software	NIH	https://imagej.net/ij/
Arivis Vision4D 3.3.0	Zeiss	https://www.arivis.com/
Arivis SIS	Zeiss	https://www.arivis.com/products/sis-converter
Columbus Analysis System	PerkinElmer	https://www.perkinelmer.com/de/lab-products-and-services/product-support.html#Columbus
CellRanger software (v7.1.0)	10X Genomics	https://www.10xgenomics.com/support
Seurat pipeline (v. 4.3.0)	Satija Lab	https://satijalab.org/seurat/
R (v. 4.2.2, with Bioconductor 3.16)	The R Foundation	https://www.r-project.org
ZEN	Zeiss	https://www.zeiss.com/microscopy/de/produkte/software/zeiss-zen.html

### Resource availability

#### Lead contact

For further information and requests regarding this manuscript, please contact the lead author, Natalia Rodríguez-Muela, at natalia.rodriguez-muela@dzne.de.

#### Materials availability

The authors declare that all results supporting the findings of this study are available within the paper and its supplemental materials. The use of the parental hiPSCs (BJ WT, 1016A, CRTD1 and SMA) and the newly generated isogenic iPSC lines is restricted by a material transfer agreement (MTA).

#### Data and code availability

• The single-cell RNA sequencing raw data is available upon approval of the Data Access Committee (DAC) on the European Genome-phenome Archive (EGA) with accession number: EGAS00001007259. All code used in this manuscript is available as of the date of publication on GitHub https://github.com/Rodriguez-MuelaLab/Grass-et-al-2024.
• This paper does not report original code.
• Any additional information required to reanalyze the data reported in this work paper is available from the lead contact upon request.

### Experimental model and study participant details

#### Patient derived iPSC lines

All experiments involving hiPSCs were performed in accordance with the ethical standards of the institutional and/or national research committees, as well as the 1964 Helsinki Declaration and its later amendments and approved by the Ethics Commission at the Technische Universität Dresden (SR-EK 80022020). The healthy BJ siPSC (HVRDi005-A code from the European Human Pluripotent Stem Cell Registry), 1016A (HVRDi007-A) and the SMA (HVRDi015-A, HVRDi017-A, HVRDi016-A) hiPSCs were kindly provided by Lee L. Rubin (Harvard University) through an MTA. The healthy CRTD1^81^ line was kindly provided by the CRTD (CRTDi004-A) Stem Cell Core Facility through an MTA. The information regarding both the SMA hiPSC lines and the healthy control lines is enclosed in.^18^^,^^37^

#### Generation of the isogenic corrected hiPSCs from SMA lines

For each of the hiPSC lines generated in this study, 1x10^6 cells were dissociated using Accutase and resuspended in 100 μL P3 nucleofection solution. Then, 2.5 μg of the pTG-Cr-SMN plasmid and 5 μg of targeting vector pTG-HR-SMN:Clover were added. Nucleofection was performed using the 4-D nucleofector system (AMAXA) and the P3 Primary Cell 4D-Nucleofector Kit (Lonza, V4XP-3024) following manufacture’s instructions (program CB-150). hiPSCs were transferred to Matrigel (Corning, 354234)-coated dishes containing (Stem Cell Technologies; 85850) with 4 μM ROCK inhibitor (Hölzel Diagnostik; S1049-50) to improve survival. ROCK inhibitor was removed 24 h post nucleofection and media was changed from there on every other day. From d2-d9 1 μg/mL puromycin (Life Technologies, A1113802) was added to select for successfully targeted cells. At day 12, the cells were nucleofected with a CRE-GFP plasmid to excise mRuby-T2A-Puromycin. 36 h post nucleofection GFP+:mRuby-cells were FACsorted and plated on matrigel-coated 10cm dishes at different clonal densities. After 10 days, 24 colonies for each line were picked, expanded and sequenced. To confirm successfully targeted *SMN2* loci, as well as presence of untargeted *SMN2* the PCR strategy shown in Figure S1B–S1C was used. Briefly, to check for untargeted *SMN2* copies forward primer upstream of the 5′ HA in combination with reverse primer in intron 7 was used. To check for targeted SMN:Clover loci the same forward primer was used, while the reverse primer was designed to bind within Clover sequence therefore only giving a product if Clover had been fused to exon 7 of the corrected *SMN2* locus. The primers used are contained in Table S1. At least four clones per line were successfully sequenced, having at least one *SMN2* copy targeted (converted to *SMN1* and Clover added in frame) and one copy that remained untargeted. Two of the successfully targeted clones per SMA line were used in this study.

#### Mouse model

All animal studies were approved by the ethics committee of the Technische Universität Dresden and the Landesdirektion Dresden (approval numbers: TVV 4/2022; 25–5131/542/6). All relevant European Union regulations, German laws (Tierschutzgesetz) and the NIH Guide for the Care and Use of Laboratory Animals (National Academies Press, 2011) were strictly followed for all animal work. Heterozygous SMNΔ7 Hb9:GFP mice (*Smn*+/−;h*SMN2*+/+; h*SMNΔ7*+/+;*Hb9:GFP*+) on an FVB background were kept under standard conditions and bred to harvest healthy (Smn+/−;h*SMN2*+/+; h*SMNΔ7*+/+;*Hb9:GFP*+) and SMA (*Smn*−/−;h*SMN2*+/+; h*SMNΔ7*+/+;*Hb9:GFP*+) mouse E10.5 embryos.

### Method details

#### Generation of the SMN:Clover vector

Briefly, one plasmid (px458, Addgene Plasmid #48138) expressing sgRNA as well as Cas9 was used to introduce double-strand breaks near Exon 7 of *SMN2* locus. A second plasmid, pTG-HR-SMN:Clover, a modified version of the commercially available plasmid HR120-PA1 (Systembio) served as targeting vector carrying *SMN1* exon 7 (C in position 6) as well as the Clover fluorophore and was used as template for homologous recombination to repair the double-strand break post cleavage by Cas9. To generate the pTG-Cr-SMN, we designed a CRISPR guide (gRNA) with an estimated cleavage site right before the stop codon of exon 7 *SMN* locus. Single-stranded (ss) oligos (IDT) listed in Table S3 were annealed and cloned into the px458 plasmid (Addgene # 48138) using BbsI and T7 DNA ligase in a one-step digestion-ligation reaction to produce the CRISPR gRNA. The correct insertion of the gRNA into the px458 plasmid, resulting in pTG-Cr-SMN, was confirmed by Sanger Sequencing (Microsynths). To generate pTG-HR-SMN:Clover, the commercially available vector HR120-PA1 (Systembio) was used as backbone. First, HR120-PA1 was digested with EcoR1 and NRU1 to remove copGFP, WPRE and PolyA. One gBlock (IDT) was then used to insert Clover coding sequence and to restore WPRE and PolyA as well as EcoR1 and NRUI restriction sites. Following another digest with EcoR1, a gBlock containing the last 372bps of intron 6 (of *SMN2*) and *SMN1* exon 7 without its stop codon - used as the 5′ homology arm (HA) and was cloned into the vector via Gibson assembly. Finally, the vector was digested with BamHI to introduce a second gBlock containing the 3′ HA -consisting of 420bp of *SMN2* intron 7 (Table S2). The successful cloning of both HAs as well as the correct sequences of the inserted gBlocks were confirmed via Sanger sequencing. The final vector also contains the double selection cassette, mRuby-T2A-puromycin, flanked by *loxP* sites, to enable preselection for successful targeted cells.

#### Validation of genome editing by PCR and Sanger Sequencing

hiPSCs were collected, washed and spun down at 135g for 5 min. Cell pellets were lysed and gDNA extracted using DirectPCR lysis reagent CELL (Viagen, 301-C) and Proteinase K (Thermo Fisher Scientific, EO0492). PCRs to confirm targeted and untargeted *SMN2* loci were performed for each of the cell lines with primer pairs shown in Table S3 using High-Fidelity 2X PCR Master Mix (NEB, M0541L) and Thermal Cycler C1000 Touch (Bio-RAD). Using the same forward primer binding upstream of the 5′ homology arm, two different PCRs were performed. First, to confirm successful targeting and therefore conversion of *SMN2* into *SMN1,* a reverse primer binding within the Clover sequence was used. Second, to confirm the presence of untargeted alleles, a reverse primer binding in intron 7 was used. Due to the distance from the forward primer, this second PCR only worked if no Clover had been inserted. To confirm successful PCRs, samples were run in 1.5% (Sigma-Aldrich, A9539) gels stained with 0.01% RedSafe (INtron Biotechnology, 21141) using Perfect Blue Gel System (Peqlab). PCR products were analyzed by Sanger sequencing (Microsynths) for final confirmation of targeted and untargeted *SMN2* alleles for each cell line.

#### Human iPSC protein turnover and proliferation assays

To determine total SMN (antibody detected), SMN:Clover and Gemin2 protein turnover, 4.000 hiPSCs per line were plated in matrigel-coated 96W plates and 2 days later and incubated with 0.5 μg/mL of cycloheximide (CHX, Sigma Aldrich, C1988) for the indicated time followed by fixation, permeabilization and immunostained. Imaging and image analysis was performed as described below. Proliferation rate of the different hiPSC lines was determined using live imaging and automated high throughput analysis. Cultures of hiPSCs (at ∼60–80% confluency) were dissociated to single cell suspensions using accutase (Corning, 25-058-Cl), washed in 1x Dulbecos PBS-Ca/-Mg (Thermo Fisher Scientific; 14190169), spun at room temperature (RT) 135g for 5 min, and plated to Black F-Bottom Greiner μClear p96 well plates (Greiner, 655090) at a density of 4.000 cells/well. The plating medium consisted of mTeSR1, 5x mTeSR1 Supplement (STEMCELL Technologies), 5% PenStep (Thermo Fisher Scientific, 1510-122), and 10μM Y-27632 (ROCKi). hiPSCs were maintained with daily media changes with mTeSR1. 2 days post plating (D2) cells were stained with 500nM of SiR-DNA (Spyrochrome, SC007) dye for 1-1.5hrs before removal and 2x 1x PBS washes. After staining cells each well was imaged in the CO2 and temperature controlled Operetta CLS (5% CO_2_ and 37°C) with 20X water immersion objective (NA 1.0; Plan Apochromat) and Alexa 647 emission filter. On subsequent imaging days, cells were stained with 500nM SiR-DNA for 30min to refresh effluxed dye and when cells reach 95–100% confluency the experiment was ended. The whole area of 10 wells in each 96w plate was imaged and quantified. To quantify hiPSC proliferation, the PerkinElmer software Harmony v4.0 was used to perform automated analysis of colony area μm^2^ at each time point. This was achieved by first applying an image filter (mean smoothing filter or sliding parabola) to clarify whole colonies in the Alexa 647 channel. SiR-DNA+ Image Regions were found on these filtered images. The resulting selected areas (μm^2^) occupied by the hiPSC colonies overtime were summed and related to the first time point measured for each of the lines.

#### hiPSC differentiation into the 3-germ layers

hiPSCs were plated in matrigel-coated 24-well plates and cultured in STEMdiff Trilineage Ectoderm, Mesoderm or Endoderm differentiation medium according to manufacturer’s instructions (STEMCELL Technologies, 5230). To measure gene expression, cells were washed 2x PBS and lysed in TRIzol. RNA isolation and mRNA expression was determined as described in the “RNA-Isolation, cDNA-synthesis and RT-qPCR” section.

#### RNA-isolation, cDNA-synthesis and RT-qPCR

hiPSCs or SCOs were washed 2x PBS. Total RNA was extracted with the TRIzol reagent (Life Technologies, 15596026), and the concentration was measured with the NanoDrop TM 1000 Spectrophotometer (Thermo Scientific). 0.5–1 μg RNA was subjected DNase I digestion using (Thermo Fisher Scientific Scientific, EN0521) according to the manufacturer’s instructions. RNA was reverse transcribed with High-Capacity cDNA Reverse Transcription Kit (Applied Biosystems, 4368814). RT-qPCR was performed with GoTaq qPCR Master Mix (Promega, A6002) and a Quantstudio5 Real-Time PCR Detection System (Thermo Fisher Scientific). mRNA expression levels were normalized to the expression of the human housekeeping gene h18S and the cycle numbers plotted (hiPSC Trilineage Differentiation) or relative values were determined with the comparative ddCT method (SCO developmental gene expression). The levels of snRNAs were measured using real-time RT-qPCR following the procedure previously described^121^ and using 5.8s as housekeeping gene. Ultimately, the gene expression was normalized against the control group of each experiment. Primers used are depicted in Table S3. 3 independent hiPSC Trilineage Differentiation experiments were analyzed. 4–8 SCOs (depending on the timepoint: 8 SCOs for d84, 6 for d18, for d28 and d38) per experiment were pooled together for RNA isolation, from 4 independent experiments.

#### Human spinal MN differentiation

hiPSCs were grown on cell culture treated dishes, coated with Matrigel, maintained with mTeSR1 and split using ReLeSR (STEMCELL Technologies, 100–0484). To induce MN differentiation, we adapted the first protocol generated from mouse ESCs, which set the ground for pluripotent cell differentiation into MNs,^47^ as we have previously used.^18^^,^^37^ Briefly, hiPSCs were dissociated and cultured in mTeSR as embryoid bodies (EBs) for 3 days in 10 cm2 ultra-low attachment dishes (ULA) (Corning, 3262). For the first 24 h mTeSR was supplemented with 10μM Y-27632 (ROCK inhibitor) and 10 ng/mL FGF-2 (Millipore, 32160702). 3 days later the media was changed to neural induction media (NIM) (day 0), containing 50% Advanced DMEM/F12 (Thermo Fisher Scientific, 12634028), 50% Neurobasal media (Thermo Fisher Scientific, 21103049), 1% Penicillin-Streptomycin (LifeTechnologies, 15140122), 1% GlutaMAX (Thermo Fisher Scientific, 35050087), 0.1mM 2-mercaptoethanol (LifeTechnologies, 21985023), 0.5x B27 (Thermo Fisher Scientific, 17504044) 0.5x N2 (Thermo Fisher Scientific, 17502048), and 20μM ascorbic acid (SigmaAldrich, A4403). From day 0 to day 4 NIM media was supplemented with 10μM SB431542 (BioTechne, 1614/10) and 100nM LDN193189 (Hölzel, M1873) to induce neural differentiation. From day 3 to day 15 1μM retinoic acid (SigmaAldrich, R2625) and 1μM Sonic Hedgehog Signaling Agonist (Millipore, 566660) were added. From day 7, 10 ng/mL BDNF (Qkine, QK050), from day 9 10μM DAPT (Tocris, 2634/10) and from day 11 10 ng/mL GDNF (Qkine, QK051) and 2μM cytosine arabinoside (AraC, SigmaAldrich, C1768) were added. On day 15–17 the EBs were dissociated with papain/DNase solution (Worthington, LK003178, LK003172) as described in^18^ and plated on 50 μg/mL poly-D-lysine (SigmaAldrich, A-003-E), 3 μg/mL laminin (Thermo Fisher Scientific, 23017015) coated plates. Media for culturing dissociated neurons was Neurobasal containing 1% Penicillin-Streptomycin, 1% GlutaMAX, 1x non-essential amino acids (LifeTechnologies, 11140050), 0.5x B27, 0.5x N2, 20μM ascorbic acid, 25μM 2-β-mercaptoethanol, 2μM AraC, 10 ng/mL BDNF and 10 ng/mL GDNF. A full media change was performed 2 days after plating, then half media changes were performed every second to third day.

#### Human MN treatment, survival assays and image analysis

When indicated, MNs were treated with 1 μM MLN4924 (Active Biochem) (to stabilize SMN protein), 0.3 μg/ml CHX (to prevent protein synthesis and therefore detect protein degradation) or DMSO control 4 days after being plated for 3 days. Cells were then fixed for 15 min in 4% PFA, permeabilized for 30 min (0.25% Triton X-(Sigma, X100) and 5% NGS (Cell Signaling, 5424S), immunostained with the indicated primary antibodies for 2h at RT and secondary antibodies for 1h at RT. Images were captured using an automated Operetta CLS microscope (PerkinElmer) with water immersion-40X magnification. Subsequent image quantification was performed using the Columbus Analysis System. MNs were identified by ISL1 fluorescence in the nucleus and nuclei were identified using Hoechst. Total SMN or SMN:Clover fluorescence intensity was determined as previously described.^37^ Briefly, after cell-identification a constant cytoplasmic region (circle) around the nucleus was defined and, by using a fully automated imager and associated software, the intensities in the SMN-immunostained and SMN:Clover channels of all the pixels in that cell region were added up giving the “total intensity” in arbitrary units. That number was then divided by the number of pixels in that region, resulting in the “mean intensity” (of a pixel in the cell) in a way that is independent of the cell size. The mean intensity per cell was averaged across 40 random fields per well and 3–5 wells per condition, containing in total hundreds to thousands of MNs in each experiment. For each experiment, three wells with no primary antibody or non-Clover cells were used to determine background fluorescence intensity. To assess survival on live MN cultures using SiR-DNA (Figures 3A–3E), after EB dissociation, neurons were plated in poly-D-lysin/laminin-coated 96 well plates (Corning, 4680) at a density of 8∗10^4^ cells/well. 3 wells per cell line were stained using the live nuclear dye SiR-DNA at 125nM for 1.5h on day 2 after plating and washed with 1X PBS. Staining of the cells was repeated every 3 days with 62.5nM SiR-DNA dye for 1h. To follow survival of the neuronal cultures, the same fields in the stained wells were imaged every 2 days using the Operetta CLS microscope with water immersion-40X objective. Image quantification was performed using the Columbus Analysis System. A size and morphology threshold was used to identify living cells and to eliminate apoptotic nuclei from quantification. To validate the increased survival of MNs derived from the most severe SMA line (Figures 3F and 3G), MNs were plated in poly-D-lysin/laminin-coated plates that were fixed 2 and 12 days later. The number of ISL1+ MNs was quantified at both time-points and the percentage of surviving MNs over that time frame for each line graphed.

#### Generation of SCOs, live imaging and size quantification

hiPSCs colonies from the 10 lines (BJ WT and the 3 isogenic trios) were accutased and 4.000 cells per well seeded into ULA 96-well plates and cultured in mTeSR for 5 days (ROCKi was added for the first 48 h). The formed stem cell aggregates were changed to neural induction media (NIM) and 3 days later embedded in 15μL Matrigel drops, transferred to ULA 10 cm dishes and kept in an orbital shaker, where the spinal cord patterning started. For the generation of ventral SCOs (vSCOs), dual SMAD inhibition (10μM SB431542 and 100 nM LDN193189) was maintained for 6 days (day 0 to day 6). On day 4 the caudalizing agent RA (1μM) and the ventralizing SAG (1μM) were added for 12 days (day 4 to day 16). The notch response inhibitor DAPT (2.5μM) was added to the culture on day 10 to enhance neural differentiation (from day 10 to day 16). Neurotrophic factors were added subsequently and until the end of the culture (10 ng/mL BDNF from day 12 and 10 ng/mL GDNF from day18). For the generation of more complex SCO containing mesodermal and neural lineages, we generated NMPs from hiPSCs^122^^,^^123^ by exposing our isogenic cohorts to the WNT agonist CHIR99021 (3μM) and FGF-2 (100 ng/mL) for the first 3 days (day 0–1 to day 3–4, respectively) prior to neuroectoderm induction. The growth factor and TGF-β family member GDF-11 (50 ng/mL) was subsequently added to the culture media, as it is naturally present during the later phases of NMP propagation *in vivo*^124^^,^^125^ and is essential for promoting the expression of lumbosacral HOX genes without hindering the expression of rostral HOX genes.^126^ Similar to our vSCO protocol, we next performed dual SMAD inhibition^127^^,^^128^ but used only LDN193189 during GDF-11 exposure (day 3 to day 6) to avoid blocking ALK5-dependent GDF-11 caudalizing effect by SB431542.^122^ An additional 3 days of LDN193189 and SB431542 treatment followed. We next added a 24 h (day 7) short pulse of the Notch signaling-inhibiting molecule DAPT (2.5μM) to enhance the generation of OLIG2+ MN progenitors and therefore increasing HB9+; ISL1+ MNs,^129^^,^^130^ and additionally from day 11 to day 17. RA (0.1 μM) and SAG were added from day 6 for 12 days. BDNF and GDNF were added at days 10 and 15 and until the SCOs were collected for analysis. To image stem cell aggregate formation and sphere growth, the 96w ULA plates were imaged two days after being seeded (day −6) in a Operetta CLS microscope, under temperature and C02 control, using a 10x air objective and imaging 10 focal planes 20μm apart, every day for 6 days. Sphere size quantification was performed blindly on images with maximum projection of all z stack sections using ImageJ software (NIH). 24–36 spheres per hiPSC line, day and experiment were measured (3–5 experiments per line).

#### SCO dissociation and labeling for multiplexed scRNA-seq

Spinal cord organoids were dissociated into single-cell suspensions using a papain/DNase solution (Worthington, LK003178, LK003172). 4–10 organoids per line (for day 4 and days 20–40, respectively) were pooled and incubated with 3mL of 1:1 0.05% Trypsin-EDTA (Life Technologies, 25300054):Papain/DNase solution for 12 min at 37°C with shaking. After enzyme inactivation, organoids were mechanically triturated through multiple pipetting rounds using 1 mL tips. Sequentially dissociated cells were transferred to NIM medium, spun down at 135g for 5 min, resuspended in fresh NIM medium, filtered with a 30-μm cell strainer and counted. 1.5 million cells per line (BJ WT, 51N-II parental and two isogenic corrected clones and 38D-I and its corrected clones) were used for TotalSeq-A antibody (Biolegend) labeling following 10X Genomics staining instructions. After the labeling, cell suspensions were spun down for 5 min 300 g at 4°C and the cell pellets were resuspended in 0.04% PBS-BSA. Cell viability was determined by microscopy using trypan blue staining (50%), and 50K cells per sample were pooled at a final concentration of 2K cells/μL in 0.04% PBS-BSA.

Droplet-based scRNA-seq was performed using the 10x Genomics Chromium Single Cell Kit v3. An aliquot of the single cell suspension was visually inspected under a light microscope to check viability and cell concentration. Single-cell suspensions concentrated at 900–2,800 cells per microliter having a viability of more than 70% were carefully mixed with reverse transcription mix and nuclease-free water according to the Chromium manual, targeting 30,000 cells per reaction. They were then loaded in a Chromium Single Cell G Chip on the 10X Genomics Chromium system.^131^ In short, the droplets were directly subjected to reverse transcription, the emulsion was broken and cDNA was purified using silane beads. After amplification of cDNA with 11 cycles using primers to enrich cDNA as well as Totalseq-A hashtag, the samples underwent SPRI bead purification, including a fractionation for smaller fragments (up to 400 bp) to enrich the hashtag sequences and larger fragments (>400 bp) to separate cDNA fragments. After quality check and quantification, the 10X Genomics single cell RNA-seq library preparation - involving fragmentation, dA-Tailing, adapter ligation and 10 cycles indexing PCR – was performed based on the manufacturer’s protocol. In parallel, the hashtag library was prepared by a 10-cycles index PCR. After quantification, both libraries were sequenced on multiple Illumina NovaSeq 6000 S4 flowcells in 200bp paired-end mode, thus generating ∼700–1200 million fragment pairs for the gene expression libraries and ∼50 million fragment pairs for the hashtag library. Raw data are available upon approval of the data access committee (DAC) on EGA with accession number: EGAS00001007259.

#### Single-cell sequencing pre-processing and data analysis

Paired-end sequencing data were pre-processed with CellRanger (10X Genomics) using a custom reference genome. To build the reference genome, the human genome (hg38) as well as gene annotation (Ensembl 104) were downloaded from Ensembl and the annotation was filtered with the `mkgtf` command of CellRanger (v6.1.2, options: `--e attribute = gene_biotype:protein_coding --attribute = gene_biotype:lincRNA --attribute = gene_biotype:antisense`). Genome sequence and filtered annotation were then used as input to the ‘mkref’ command of CellRanger to build the appropriate CellRanger reference. A reference for the TotalSeq cell multiplexing antibodies was also manually created for CellRanger. The raw sequencing data was then processed with the `multi` command of the CellRanger software (v7.1.0) provided by 10X Genomics with standard parameters. For data analysis, Cell Ranger demultiplexed count matrices were loaded in R (v. 4.2.2, with Bioconductor 3.16) and processed according to the standard Seurat pipeline (v. 4.3.0^132^). First, the entire dataset including all three time points was filtered for low-quality cells (nFeatures >1000 and percent of mitochondria genes <10%), normalized, scaled and dimensional reduction was calculated using the standard Seurat functions. For normalization, the gene expression values were normalized by total UMI counts per cell, multiplied by 10,000 (TP10K) and then log transformed by log10 (TP10k+1). Subsequently, the data was scaled and centered. For dimensionality reduction, PCA was performed on the top 2,000 variable genes identified using the vst method. For two-dimensional representation of the data structure, uniform manifold approximation and projection (UMAP) non-linear dimensionality reduction was calculated using the first 20 principal components (PCs). Identical data pre-preprocessing and dimensionality reduction was performed when subsetting the dataset for the specific time points. Next, for each time point, cells were clustered using the Louvain algorithm based on the first 20 PCs and a resolution of 0.1. Cluster-specific marker genes were calculated with the Wilcoxon rank-sum test using the FindAllMarkers Seurat function (parameters: min.pct = 0.20, logFC.threshold = 0.20). The biological identity of each cluster was annotated by combining Enrichr analysis web server with the manual annotation of the cluster-specific marker genes. All genes expressed in each cell cluster with a p_adjusted value equal to 0 (ranging from 60 to several hundred depending on the cluster) were used in the “Human Gene Atlas” and “ARCHS4 Tissues” databases of Enrichr-Cell Types function to identify the cluster identity. Annotation of cell clusters was further determined by gene expression of known markers crosschecked with PanglaoDB database and published datasets.^88^^,^^133^^,^^134^ Finally, clusters annotated to the same cell type were merged to a single metacluster of cells. Confusion matrix was calculated as the frequency of cells from each group in each cluster. All data were visualized with using standard Seurat functions and custom ggplot2 (v. 3.4.0) functions. All code used in this manuscript will be made available upon acceptance on GitHub (https://github.com/lorenzobonaguro/Grass_et_al_2023_SMA). Seurat objects and processed data files will be available upon request. To ensure reproducibility the entire analysis was performed within a containerized environment including all required packages and functions (jsschrepping/r_docker:jss_R422_bioc316_v2). The docker image is available at hub.docker.com.

#### Cryosectioning, IF, imaging and image analysis

After overnight fixation in 4% PFA, SCOs were washed o/n in 1x PBS and cryopreserved gradually in sucrose before being embedded (TissueTek OCT, Sakura, 4583) and flash-frozen on dry-ice. The cryoblocks were sectioned at 15μm thickness using the Cryostat Leica CM3050S (Leica). Sections were collected onto SuperFrost Plus Slides (Thermo Scientific) and kept at −80°C. For immunofluorescence staining of SCO sections, the slides were post-fixed in 4% PFA for 10 min, washed and blocked for 1h with Blocking Buffer (BB) (0.25% Triton X-, 5% NGS, 0.1% Tween 20 in 1x PBS) at RT in a humid chamber. Primary antibodies diluted in BB were added for 2h at RT. Slides were washed and secondary antibodies added for 1h at RT. After several washes, Hoechst33342 (Life Technologies, H3570) was added and the slides mounted on coverslips with Vectashield Antifade Mounting Medium (Biozol Diagnostica, VEC-H-1000). Imaging was carried out using a Zeiss Confocal Spinning Disc microscope using multi-slide holders and automatic scan of tile regions, with a 20x PlanApoChromat Air Objective for all developmental times of vSCOs and neuromuscular organoids, except for day 40, for which 10x Zeiss Plan-Apochromat Air objective and 3x3 binning was used due to the large organoid sizes. z stack scans of 15μm thickness with an interval of 1μm were recorded for each organoid section. The same imaging parameters were used across all SCOs derived from all hiPSC lines for each of the antibodies used. Maximum intensity projections of z-Stacks were made and stitched with 10% tile overlap by Hoechst33342 as reference channel with Global Optimizer option, using ZEN 3.2 blue (Zeiss). Automatic quantification of the number of cells positive for the marker of interest was carried out in Arivis Vision4D 3.3.0 (Zeiss). Briefly, CZI.files were converted into arivisSIS format with no compression using Arivis SIS Converter (Zeiss). The quantification pipeline in Arivis Vision4D 3.3.0 included: 1) a fluorescence threshold to exclude background (based on 2ry-only stained SCOs), 2) global enhancement "Simple Sharpening Filter", 3) BlobFinder-nuclear segmentation tool for Hoechst33342-Channel and 4) segment operation "Feature-Filter" on surface-area (μm^2^) to remove small artifacts. Quantification was carried out using "Batch Analysis" for all SCOs. 3–4 SCOs per experiment were analyzed from 3 to 6 independent experiments. For mouse embryo histology, control (Smn+/−;h*SMN2*+/+; hSMNΔ7+/+) and SMA embryo (Smn−/−;h*SMN2*+/+; hSMNΔ7+/+) littermates were harvested at embryonic day E10.5 and fixed in 4% paraformaldehyde in PBS overnight (o/n), and cryopreserved in 15–30% sucrose gradients over two days before OCT embedding. For evaluating the relative area occupied by the developing neural tube vs. mesodermal tissues, 4 15μm thick coronal sections of the lumbar segments of the elongating spinal cord surrounding the developing hind limbs were used. Quantification of the areas occupied by the neural tube (identified anatomically and based on SOX2 expression) or mesodermal tissues were performed using the ImageJ Measure plugging. For quantifying the percentage of NMPs, all lateral sections (15μm thick) of the most caudal segments containing the tailbuds regions were used. Immunostaining and imaging of these sections was conducted as previously described for SCO cryosections. Images were cropped to only include the tailbud region for quantification, somites anterior to the tailbud were excluded. The total number of nuclei in the tailbud and NMPs positive for SOX2 and TBXT were counted using automated ImageJ macros and the average of the percentage of NMPs of all imaged sections for each embryo was calculated accordingly. The genotype of the samples was kept blind for the experimenter. Picrosirius Red staining was used to collagen fibers. SCO cryosections were fixed for 10min in 4%PFA after thawing, rehydrated for 10min in 1xPBS, washed 2 × 5min in ddH20 and stained with Giemsa’s Hematoxylin for 1 min at RT. Next, slides were de-stained 2 × 5min in ddH20 and treated with Picrosirius Red Staining Solution (made by dissolving Direct Red 80 (Sigma-Aldrich, 365548-5G) in Picric Acid Solution (Sigma-Aldrich, P6744-1GA), according to the manufacturers recommendations) for 1h at RT and subsequently de-stained 2 × 5min in acetic ddH20 (1% Glacial acid), counterstained for 30s with alcoholic Eosin-solution at RT and repeatedly dipped into water until Eosin stopped de-staining from the slide. Tissue was sequentially dehydrated in 50/70/90/95/100% EtOH and 2 × 5min in Xylene, before slides were mounted in Entellan (Merck, 107960). Imaging of one axial plane per SCO was carried out using ZeissApotome2 and a 10x objective. Images were stitched with 15% Tile-Overlap and further edited by applying an Unsharp Mask Filer. Picrosirius Red+ areas were quantified using ImageJ Measure plugin in 3–4 SCOs per line and experiment and from N = 4–5 independent experiments. The quantifications were performed blinded to the identity of the lines.

#### Co-immunoprecipitation assay

hiPSCs were lysed in lysis buffer (25mM Tris HCl pH 7.4, 62.5mM NaCl, 2% NP-40 (Thermo Fisher Scientific Scientific 85124), 1mM EDTA (Thermo Fisher Scientific, 15575020), 100x HALT protease inhibitor cocktail (Thermo Fisher Scientific, 1861278), and 0.03 U/μL DNase in 1x PBS). Cells were lifted from the plate using ReLeSR (Stem Cell Technologies; 100–0484) and hiPSCs were spun for 5 min at RT and 135g. Samples were placed on ice and mechanically lysed in pre-chilled lysis buffer using pre-chilled pipette tips and passing through pre-chilled syringes 10 times. Lysate was incubated on ice for 20min before spinning down for 10 min at 13.523g and 4°C. 1 mg of protein was used in each reaction, and total lysate was taken from each sample prior to equalizing the reaction volume to 500μL with Co-IP wash buffer (lysis buffer without Protease Inhibitor or DNase). 15μL of GFP-Trap DynaBeads (Chromotek, gtd-20), 2μg anti-SMN antibody or 0.1μg anti-IgG mouse (used as control) were added to each reaction. Reactions were incubated overnight at 4°C with rotation, and then 15μL of Pierce Protein A/G Magnetic Beads (Thermo Fisher Scientific, 88803) were added to the anti-SMN and anti-IgG reactions and incubated for 2 h at 4°C with rotation. Following bead incubation, beads were separated from supernatant and washed 3x with pre-chilled wash buffer and 3x with pre-chilled 1x PBS for 10min each at 4°C with rotation. Following washes, proteins were eluted from beads with 2x Laemli Buffer (Bio-RAD, 1610737) with 2-mercaptoethanol and boiled at 95°C for 15min along with total lysate samples. Samples were run on western blot following the described procedure below and proteins were probed for the indicated antibodies.

#### Western Blot

Cells lysed in RIPA buffer (Thermo Fisher Scientific, 89900) with complete protease and phosphatase inhibitors as described above. Western blots were performed using AnykD Criterion TGX Precast Midi Protein Gels (Bio-RAD, 5671124, 5671123). Gels were run in 1x Tris-Glycine SDS Running Buffer (Thermo Fisher Scientific, LC26755) using 60V for ∼30min and 100-110V for ∼2h. After running, the gel was equilibrated in 1x Tris/Glycine Transfer Buffer (Bio-RAD, 1610734) for ∼5min. Proteins were transferred from gel to pre-made TransBlot Turbo-Transfer Pack (Bio-RAD, 1704157) in the semi-dry Trans-Blot Turbo System (Bio-RAD, 1704150). Once transferred, the membrane was rinsed with diH_2_O and stained with Ponceau to confirm even loading. Membranes were then blocked with 5% nonfat dried milk powder (PanReac Applichem ITW Reagents, A0830) in 1x TBST (ChemCruz, sc362311) for 1 h at RT and agitation. The desired antibody was then added and incubated at 4°C overnight; the next day the membrane was washed 3x with 1x TBST for 10min each, probed for the secondary antibody diluted in 5% milk at RT with shaking, and washed again. The signal was developed using western blot substrate (SupraSignal West PICO PLUS Chemiluminescent Substrate; Thermo Fisher Scientific, 34580), X-ray Films (FUJI, 4741019289), and the Cawomat 2000 IR X-ray developer. Densitometric analysis was performed on scanned autoradiographs using the Quantity One software (Bio-RAD).

#### Primary antibodies used

anti-SMN (for co-IP) (Novus Biologicals, NB100-1936); anti-IgG mouse (Santa Cruz, sc2025); anti-SMN (for immunofluorescence) (BD Biosciences, 610646); anti-Gemin2 (Abcam, ab150383); anti-SOX2 (Invitrogen, 14-9811-82); anti-NESTIN (Millipore, MAB353); anti-ISL1 (Abcam, ab109517); anti-NKX6.1 (Developmental Studies Hybridoma Bank, F55A10); anti-MAP2, NovusBiologicals NBP2-25156); anti- TBXT (Cell Signaling, 81694); anti-ACTA2 (Sigma-Aldrich, A2547); anti-CHAT (Proteintech, 20747-1-AP); anti-SMI32/anti-Neurofilament H (NF-H) (Biolegend, 801701); anti-Desmin (Abcam, ab15200); anti-Titin (Novus Biologics, NB600-1206); anti-β-Actin (Cell Signaling, 8H10D10), anti-β-Tubulin (Cell Signaling, 2146).

### Quantification and statistical analysis

Statistical significance was determined using GraphPad Prism 10.0.3 (Graphpad Software, Inc.). To test the Gaussian distribution of residuals Shapiro-Wilk test was performed. To test equal distribution of standard deviations (SD) Bartlett’s test was performed. If no Gaussian distribution of the residuals, nonparametric Kruskal-Wallis test was performed. If no equal SD among groups, mixed Brown-Forsythe and Welch ANOVA tests were performed. One-way ANOVA was performed for datasets composed of only one variable (e.g., genotype) and two-way ANOVA was chosen for datasets composed of two variables (e.g., genotype and time during differentiation). Unpaired two-tailed t-Test was used for the *in vivo* analysis. “N” indicates the number of independent biological experiments; “n” indicates the number of technical replicates (i.e., individual spheres or organoids per experiment). For all organoid-containing assays 3–6 independent experiments were performed (N), each one consisting of 3–5 organoids (n). A confidence interval of 95% was used for all comparisons. Graphs indicate mean + SEM. ∗*p* < 0.05, ∗∗*p* < 0.01, ∗∗∗*p* < 0.005, ∗∗∗*p* < 0.001, ∗∗∗∗*p* < 0.0001, ns, non-significant.

## Supplemental information

Document S1. Figures S1–S11 and Tables S1–S3

Document S2. Article plus supplemental information
