# Supplementary material for: Isogenic patient-derived organoids reveal early neurodevelopmental defects in spinal muscular atrophy initiation
Source: Cell Rep Med. 2024 Jul 26;5(8):101659. doi: 10.1016/j.xcrm.2024.101659 (PMC11384962; doi:10.1016/j.xcrm.2024.101659)
Supplement: Document S1. Figures S1–S11 and Tables S1–S3 [file mmc1.pdf]

Cell Reports Medicine, Volume *5*

Supplemental information

Isogenic patient-derived organoids reveal early neurodevelopmental defects in spinal muscular atrophy initiation

Tobias Grass, Zeynep Dokuzluoglu, Felix Buchner, Ines Rosignol, Joshua Thomas, Antonio Caldarelli, Anna Dalinskaya, Jutta Becker, Fabian Rost, Michele Marass, Brunhilde Wirth, Marc Beyer, Lorenzo Bonaguro, and Natalia Rodriguez-Muela

SUPPLEMENTARY MATERIAL

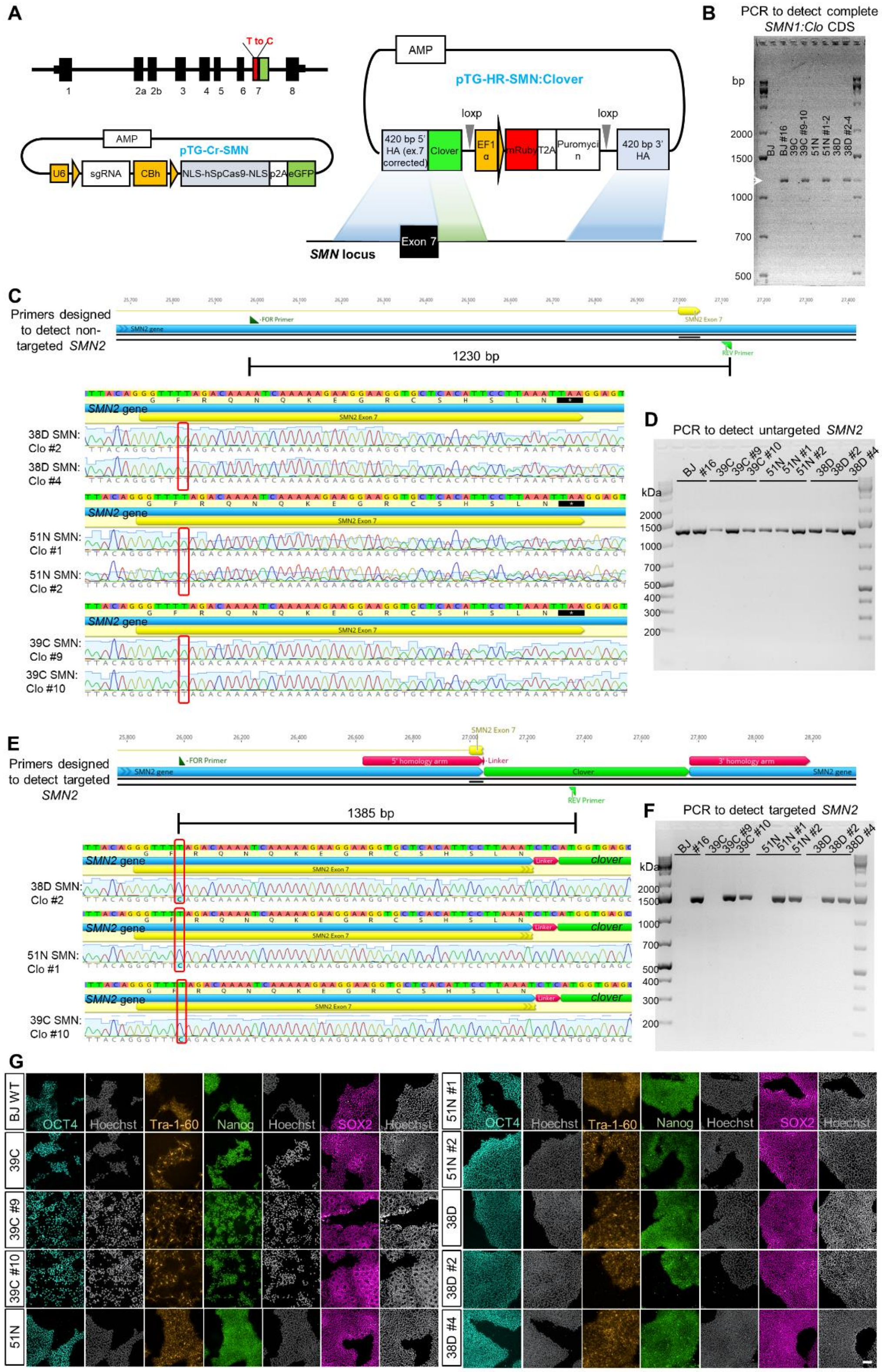

Supplemental Figure 1. Generation of isogenic control hiPSCs from various severities of SMA human iPSC lines, related to Figure 1. (**A**) Scheme of pTG-HR-SMN:Clover targeting vector used for knock-in mutagenesis to generate the isogenic hiPSC lines from the SMA parental lines. (**B**) Electrophoresis gel showing the PCR products and expected band sizes (~1156 bp) of cDNA from the parental and genome-edited lines spanning exon1 of *SMN1/2* and reverse primer through the clover sequence. (**C**) In silico design to detect a non-targeted *SMN2* copy in a parental SMA line and chromatograms from

the targeted lines showing a non-edited *SMN2* copy. *SMN2* locus is indicated by the blue bar. Exon7 of *SMN2* is labeled by a yellow bar. The primers used for PCR amplification covering the regions are indicated by the green triangles and the sizes of the resulting gDNA fragments are indicated below. (**D**) Electrophoresis gel showing the PCR products corresponding to primer pair shown in (C) for the healthy control untargeted BJ WT hiPSC line as well as targeted BJ WT, all 3 untargeted SMA parental lines as well as two targeted *SMN1*:Clover clones per line. The gel shows that for each line at least one *SMN2* locus remained unedited. (**E**) In silico design to detect a successfully targeted *SMN2* copy in a parental SMA line and chromatograms from the targeted lines showing an edited *SMN2* copy. *SMN2* locus is indicated by the blue bar. Exon7 of *SMN2* is labeled by a yellow bar. The 5' and 3' gBlocks used for the homologous recombination are indicated by pink bars, the Clover sequence (which is part of the targeting vector) is shown in green. The primers used for PCR amplification covering the region are indicated by the green triangles and the size of the resulting gDNA fragments is indicated below. (**F**) Electrophoresis gel showing that for each of the clones at least one *SMN2* locus has been successfully targeted and converted to *SMN1*:Clover. (**G**) Representative images from the hiPSCs used in the study fixed and immunostained against the pluripotency markers OCT4 (cyan), SOX2 (magenta), Tra-1-60 (orange) and NANOG (green). Nuclei were identified with Hoechst. Scale bar, 50 µm.

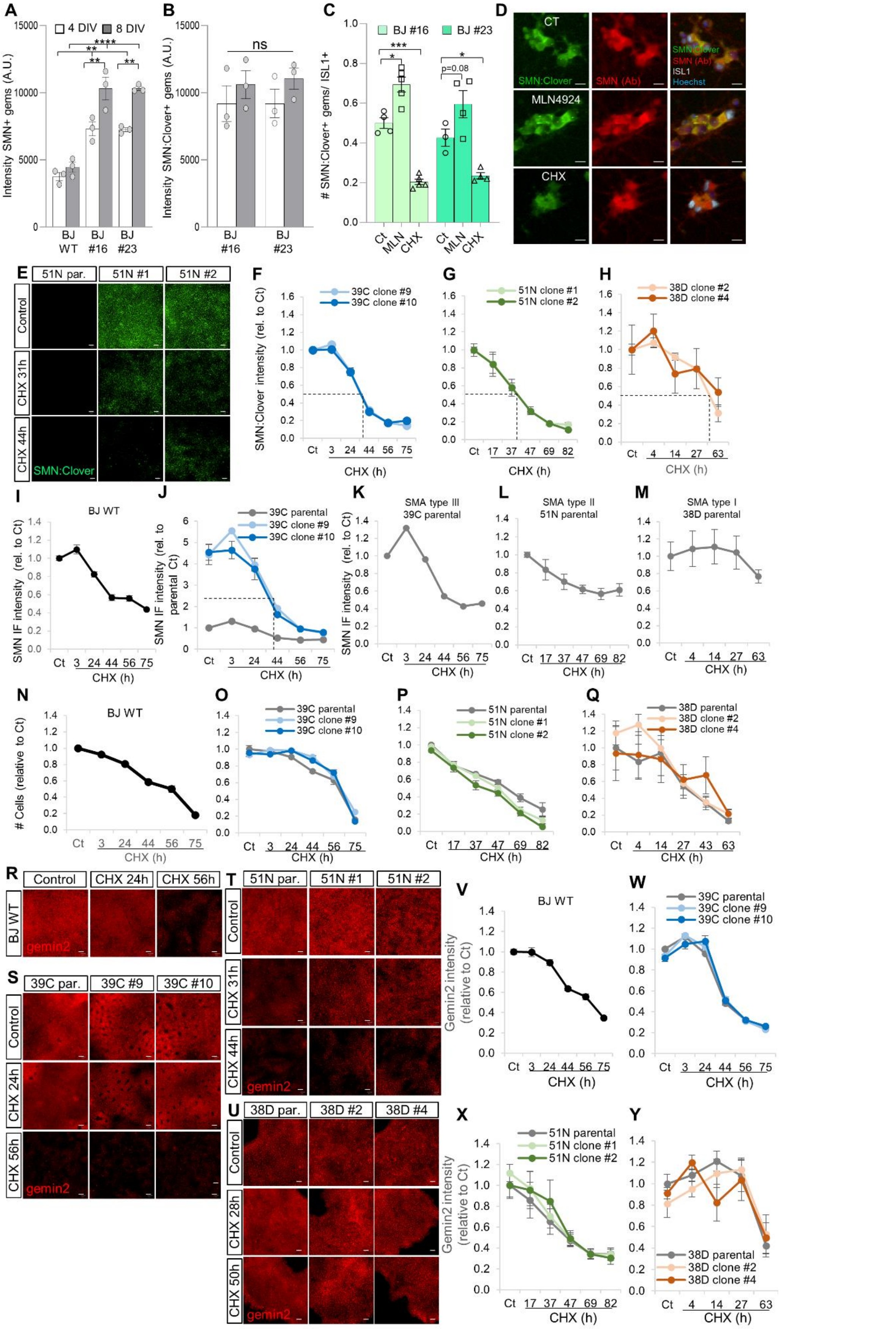

Supplemental Figure 2. **The Clover reporter tagging SMN C- terminus does not alter the biology of the protein, related to Figure 1.** (**A**) Quantification of the fluorescence intensity of nuclear gems endogenously identified by SMN:Clover or

immunostained using an anti-SMN antibody (**B**) in MN cultures generated from the healthy BJ WT line and two BJ SMN:Clover clones (“BJ #16” and “BJ #23”), 4 and 8 days after being plated (One-way ANOVA/ Tukey’s multiple comparison test, n=3). (**C**) Quantification of the number of SMN:Clover+ nuclear gems per ISL1+ MNs in MN cultures generated from two BJ SMN:Clover edited clones 4 days after being plated with 1 µM MLN4924 or 0.3µg/ml CHX (*p<0.05 One-way ANOVA followed by Tukey’s multiple comparison test, n=4). Representative images are shown in (**D**) (SMN, red; ISL1, cyan; Hoechst, blue; endogenous SMN:Clover, green). Scale bar, 10 µm. (**E**) Representative images of endogenous SMN:Clover from the isogenic SMA type II hiPSCs trio (51N parental line and corrected clones #1 and #2) treated with CHX versus control (DMSO). Scale bar, 50 µm. Quantification of the SMN:Clover fluorescence intensity (proxy for protein levels) in the isogenic corrected 39C-III (**F**), 51N-II (**G**) and 38D-I (**H**) clones upon CHX treatment. Quantification of the total SMN fluorescence intensity detected by antibody labeling in hiPSCs treated with CHX for the indicated times (in hours) relative to the DMSO-treated cells (Ct) to determine total SMN protein (Clover-tagged and untagged) half-life in the healthy control BJ (**I**), SMA type III isogenic trio (**J**) and the parental SMA type III (**K**), type II (**L**) and type I (**M**). Quantification of the number of hiPSCs treated with CHX for the indicated times (in hours) related to the DMSO-treated cells to determine cell toxicity caused by the treatment in the healthy control BJ (**N**) SMA 39C-III and its corrected clones (**O**), 51N-II and its corrected clones (**P**) and 38D-I and its corrected clones (**Q**). (**R**) Representative images showing immunostaining against Gemin2 in healthy BJ hiPSCs, (**S**) SMA 39C-III and its corrected clones, (**T**) SMA 51N-II and its corrected clones and (**U**) SMA 38D-I and its corrected clones after treatment with CHX for the indicated times or vehicle (Control, DMSO). Scale bar, 50 µm. (**V-Y**) Quantifications of Gemin2 immunofluorescence intensities of the respective lines upon the treatments.

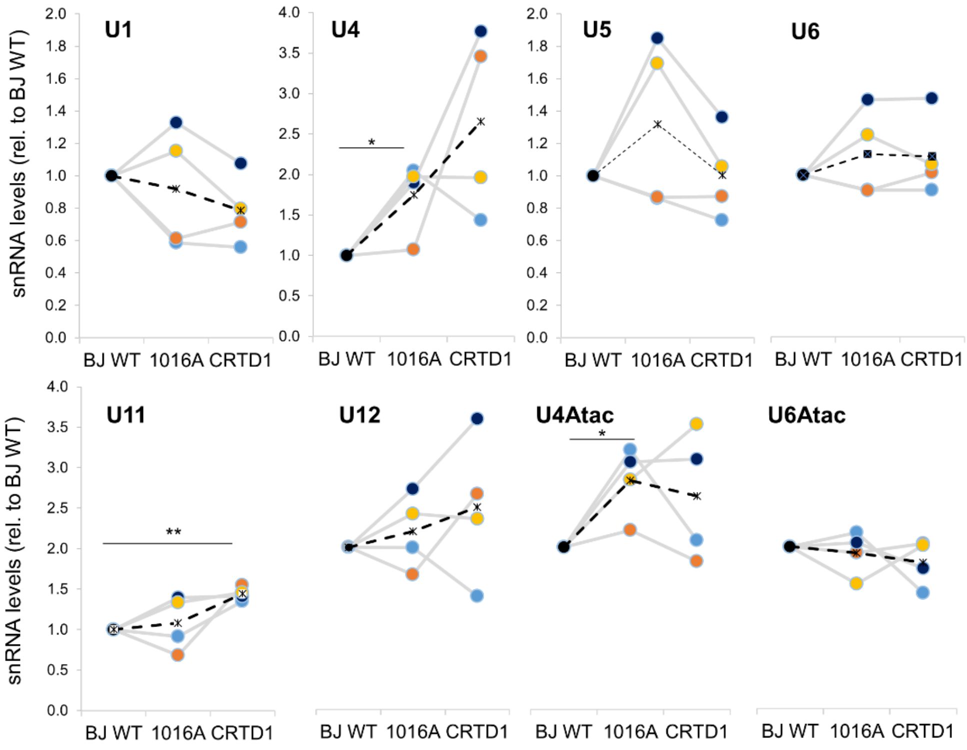

Supplemental Figure 3. **Minor and major spliceosome snRNAs show similar expression levels across three healthy control iPSC lines, related to Figure 1.** RT-qPCR analysis of snRNAs from EBs derived from the healthy control BJ WT, 1016A and CRTD1 iPSCs. RNA levels are expressed relative to BJ WT levels for each individual experiment (color-coded; One-way ANOVA/Fischer's LSD multiple comparison test, N=4). Dotted line indicates the average values for all experiments.

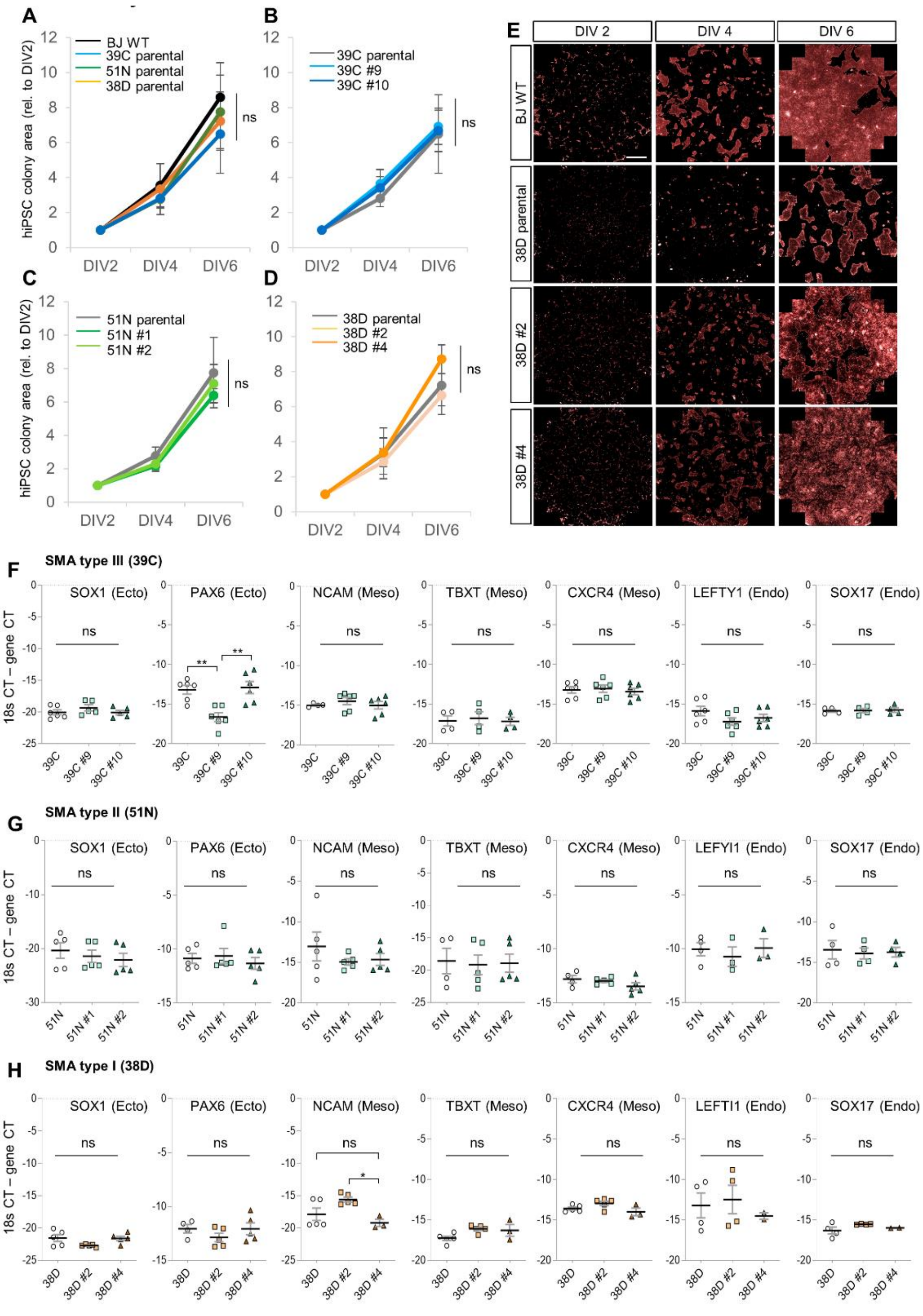

Supplemental Figure 4. **The corrected isogenic SMA hiPSCs proliferate and differentiate into the three-germ layers similarly to the SMA parental lines and healthy control, related to Figure 1.** Quantification of hiPSC colony growth over time. Colony area growth of BJ WT compared to the 3 SMA lines (**A**), 39C-III compared to its 2 isogenic clones (**B**), 51N-II compared to its 2 isogenic clones (**C**) and 38D-I compared to its 2 isogenic clones (**D**) (Two-way ANOVA/Tukey's multiple comparison test, N=3-4). (**E**) Representative images showing SiR-DNA-labeled hiPSC nuclei from the BJ WT and SMA 38D-I and its corrected clones after being plated for 2, 4 and 6 days. Scale bar, 1 mm. See also Figure S5A-B. mRNA expression qPCR quantification of ectodermal, endodermal and mesodermal marker genes from hiPSC SMA type III (**F)**, type II (**G)** and type I (**H**) and their isogenic corrected clones cultured in STEMdiff™ Trilineage Differentiation Kit. Graphics represent CT mRNA expression levels of the indicated genes subtracted from the housekeeping gene (18s) expression levels (One-way ANOVA/Tukey's multiple comparison test, N=4-6). See also Figure S5C-P.

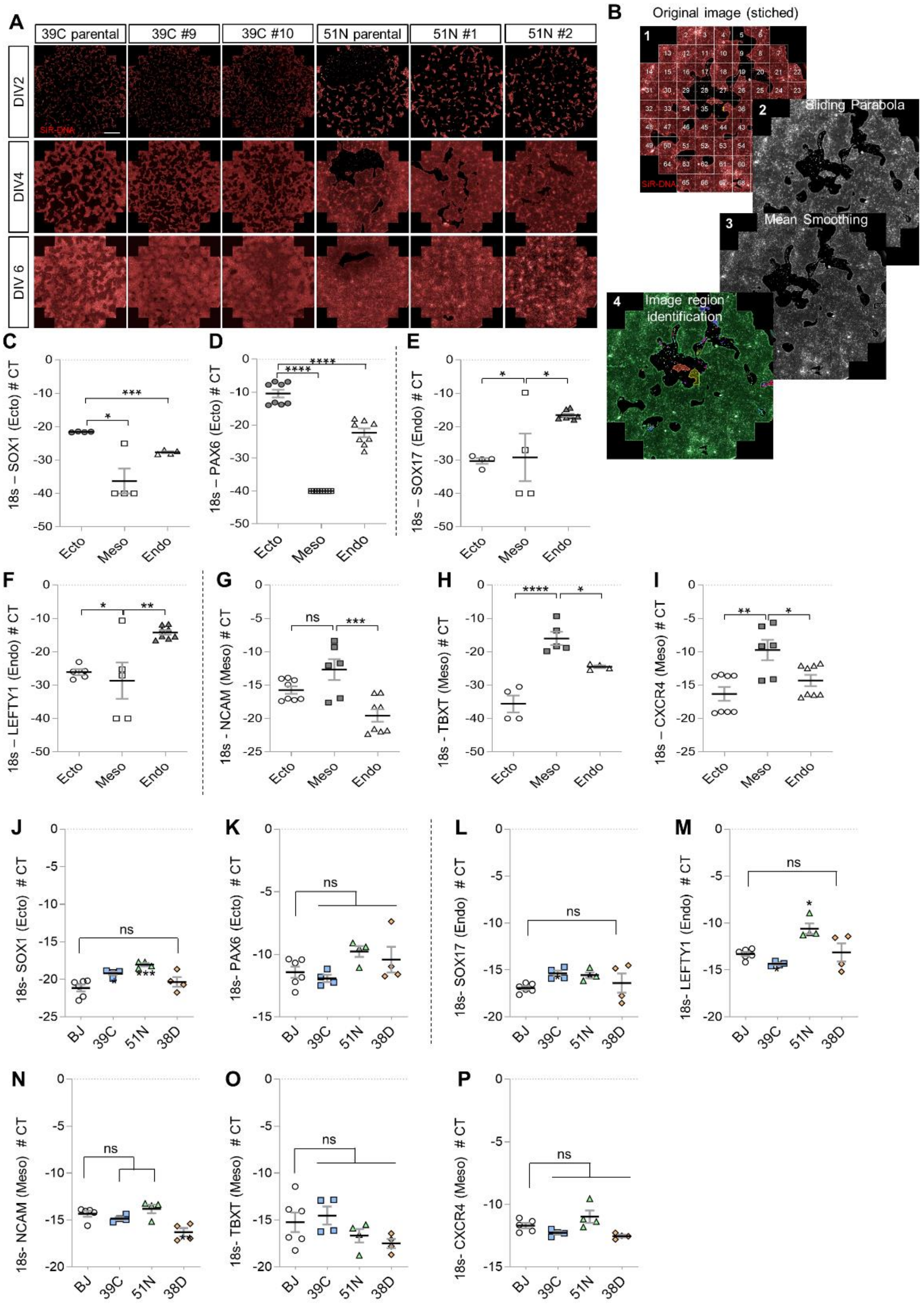

Supplemental Figure 5. **The corrected isogenic SMA hiPSCs differentiate into the three-germ layers similarly to the SMA parental and healthy control hiPSC lines, related to Figure 1. (A)** Representative images showing SiR-DNA labeling of type III and II isogenic iPSC trios 2, 4 and 6 days after being plated in Matrigel-coated 96w plates. Scale bar, 1 mm. (**B**) Exemplification of the image processing steps done by the Harmony v4.0 software script to detect the SiR-DNA labeled hiPSCs from all imaged wells in each well (1), filter the images by applying a Sliding Parabola algorithm (2) or a Mean Smoothing algorithm (3) and then identify the area occupied by the whole Image Region (4). qPCR quantification of expression markers of ectodermal *SOX1* (**C**) and *PAX6* (**D**), endodermal *SOX17* (**E**) and *LEFTY1* (**F**) and mesodermal *NCAM* (**G**), *TBXT* (**H**) and *CXCR4* (**I**) markers from BJ WT hiPSCs cultured in STEMdiff™ Trilineage Differentiation Kit (STEMCELL). Graphics represent CT mRNA expression levels of the indicated genes subtracted from the housekeeping gene (18s) expression levels. The colored symbols in each graph represent the specific medium-cultured hiPSCs where the highest expression of that gene is expected (One-way ANOVA/Tukey's multiple comparison test, n=4). qPCR quantification of ectodermal *SOX1* (**J**) and *PAX6* (**K**), endodermal *SOX17* (**L**) and *LEFTY1* (**M**) and mesodermal *NCAM* (**N**), *TBXT* (**O**) and

CXCR4 (**P**) markers from BJ WT and SMA hiPSCs cultured in STEMdiff™ Trilineage Differentiation Kit (see Materials and Methods). Graphics represent CT mRNA expression levels of the indicated genes subtracted from the housekeeping gene (18s) expression levels (One-way ANOVA/ Tukey's multiple comparison test, n=4).

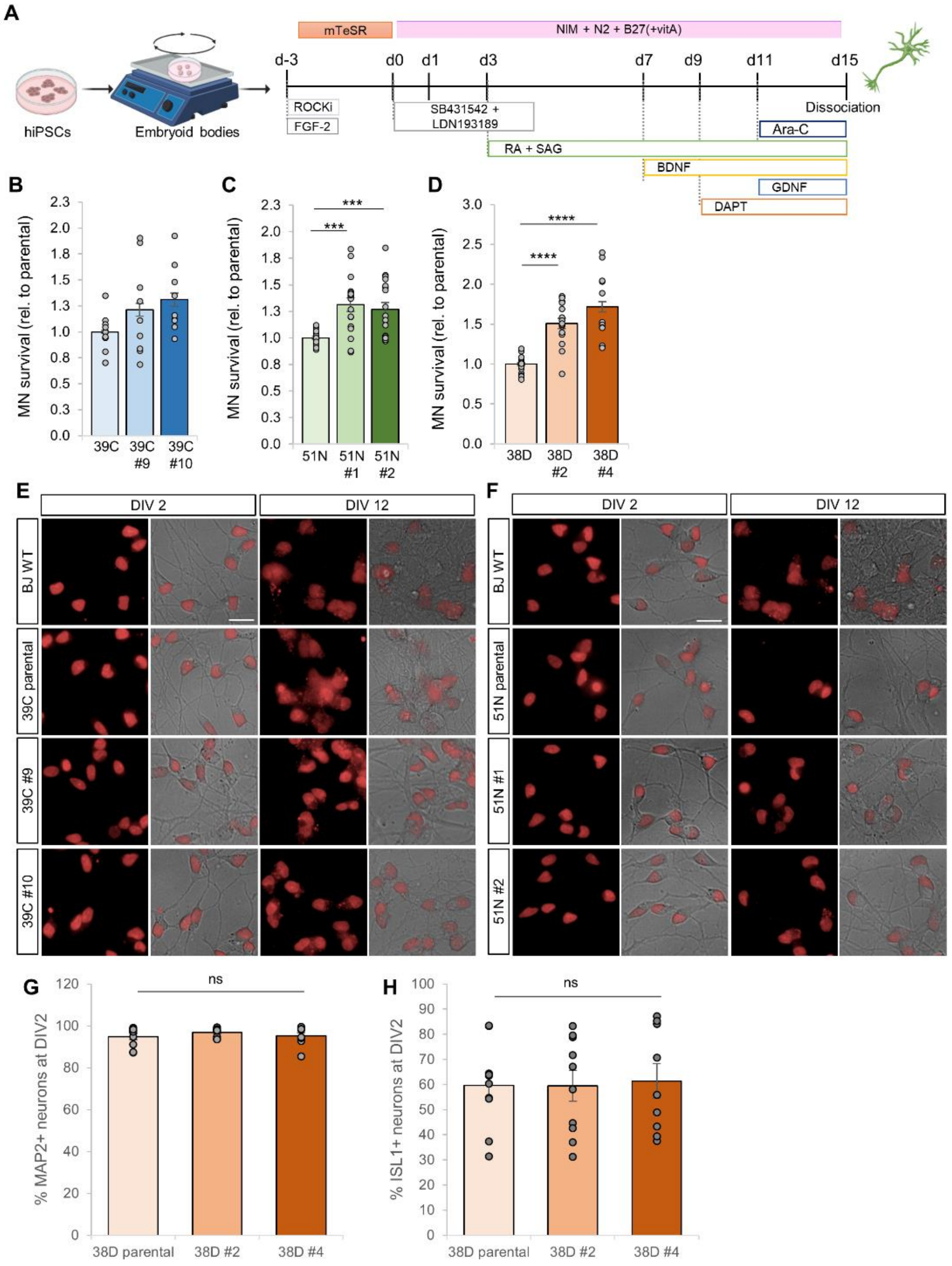

Supplemental Figure 6. **Increased MN survival and similar percentage of ISL1+ and MAP2+ cells in the isogenic corrected clones compared to the parental SMA type I line, related to Figure 2.** Schematic representation of the protocol followed to generate hiPSC-derived spinal MNs (**A**). Quantification of the percentage of hiPSC-derived neurons that survive 10 days after being plated in 96w plates. Two days after plating the MN cultures were labeled with SiR-DNA and imaged live (Operetta CLS). The same fields and wells were imaged again after 10 days and the percentage of surviving neurons was quantified. The graphs show these values as a percentage of survival of the type III isogenic corrected cultures relative to the 39C-III parental (**B**), the type II isogenic corrected cultures relative to the 51N-II parental (**C**) and the type I isogenic corrected cultures relative to the 38D-I parental (**D**). Representative images of BJ WT, SMA types III (**E**) and II (**F**) and both their respective isogenic corrected iPSC-derived MN cultures stained with SiR-DNA (red) after 2 and 10 days in culture. Bright field images of the corresponding neurons are also shown. Scale bar 20 µm. (**G**) Percentage of MAP2+ cells and ISL1+ MNs (**H**) in the MN cultures derived from the SMA 38D-I and both isogenic corrected hiPSC lines 2 days after being plated (One-way ANOVA/ Tukey's analysis, n=10).

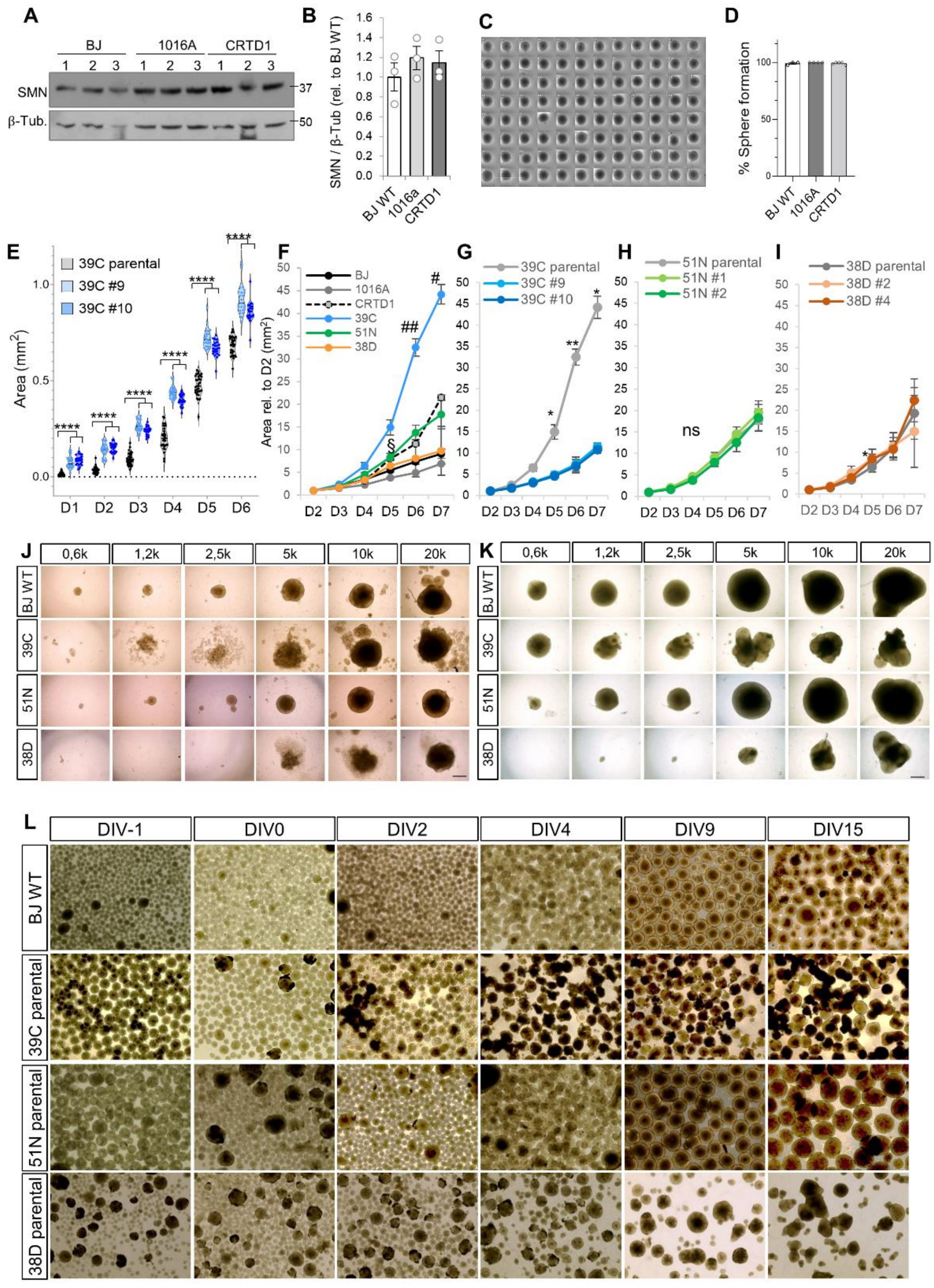

Supplemental Figure 7. **SMA hiPSCs are less efficient at self-assembling into spheres than the isogenic corrected clones or healthy control lines, related to Figure 3.** (**A**) Representative western blot from hiPSC lysates from the 3 healthy control lines used in this study showing SMN and quantification (**B**) (One-way ANOVA/Tukey's multiple comparison test, N=3). (**C**) Representative ULA 96w plate containing one stem cell aggregate per well 7 days after 4.000 51N-II SMA hiPSCs were seeded to illustrate the robustness of the culture. (**D**) Quantification of the percentage of hiPSC-seeded wells that formed a sphere two days after seeding for the healthy control lines. (**E**) Quantification of the area (mm^2) of SMA 39C-III and isogenic corrected hiPSC-derived spheres 1 day after the hiPSCs were seeded and until day 6. The violin plot exemplifies the size distribution of the individual spheres quantified for one representative experiment (Two-way ANOVA/ Tukey's analysis, n=30 spheres). (**F**) Quantification of the WT and SMA hiPSC-derived sphere size (mm^2) growth over time representing the average of at least 30 spheres imaged and quantified per line and per experiment (N=4-8 experiments) relative to the average sphere size at day 2 for each of the lines. Quantification of sphere growth for (**G**) 39C-III parental and isogenic corrected clones, (**H**) 51N-II and isogenic corrected clones and (**I**) 38D-I and isogenic corrected clones (Two-way ANOVA/ Tukey's

analysis; statistical significance between the BJ WT and the SMA lines -F- or the SMA and the corrected clones -G,H,I- is shown. # represents comparisons between BJ WT and 39C-III; § between BJ WT and 51N-II, N=3-6 and at least 30 spheres per experiment). (**J**) Representative images of the self-assembled spheres generated from BJ WT and the SMA hiPSCs 5 days after seeding. An increasing number of hiPSCs was seeded as a single-cell suspension in ULA 96w plates to determine efficiency of the hiPSCs to self-assemble depending on the starting cell number. Scale bar 400 µm. (**K**) Same spheres as shown in (J) 10 days later. (**L**) Representative images of embryoid bodies (EBs) generated following the protocol detailed in Figure S6A. hiPSCs were seeded as a single-cell suspension in ULA 10cm^2 dishes.

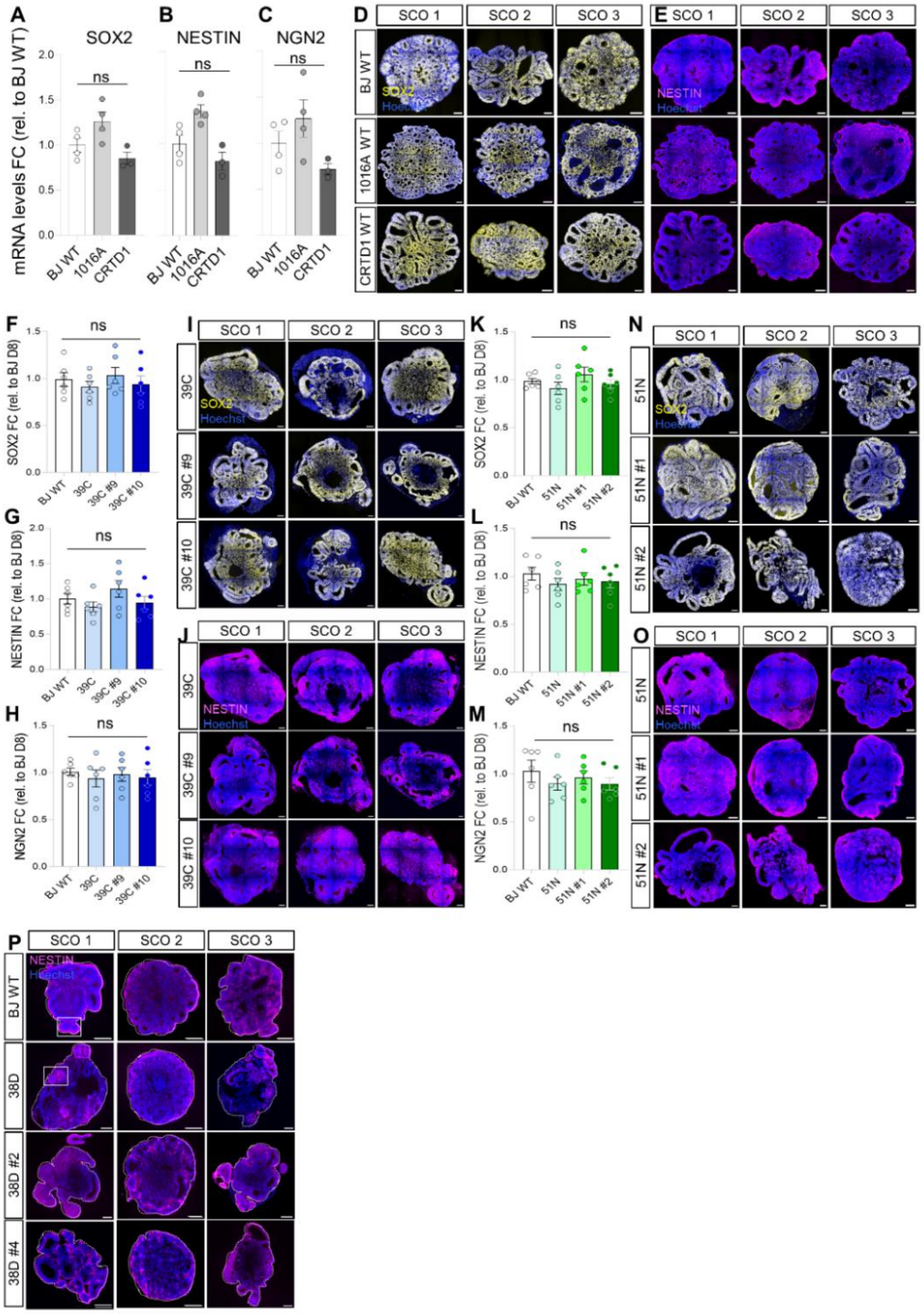

Supplemental Figure 8. **No major early neurogenesis defects are observed in the mild SMA vSCOs compared to isogenic and healthy controls, related to Figure 4.** mRNA expression qPCR quantification of *SOX2* (**A**), *NESTIN* (**B**) and *NGN2* (**C**) from day 8 vSCOs derived from the three healthy control lines used in this study (One-way ANOVA/ Fisher's LSD multiple comparison test, N=4, 4-8 pooled vSCOs per experiment) and representative images showing SOX2 (yellow) (**D**) and NESTIN (**E**). Scale bar, 100 µm. mRNA expression qPCR quantification of *SOX2* (**F**), *NESTIN* (**G**) and *NGN2* (**H**) from day 8 vSCOs derived from the 39C-III isogenic trio versus BJ WT (One-way ANOVA/ Fisher's LSD multiple comparison test, N=4, 4-8 pooled vSCOs per experiment) and representative images showing SOX2 (**I**) and NESTIN immunostaining (**J**). Similar qPCR and immunostaining analysis from the 51N-II isogenic trio (**K**-**M** and **N**-**O**, respectively). (**P**) Representative images from day 8 vSCOs derived from the BJ WT and the 38D-I isogenic trio showing NESTIN (magenta). Nuclei stained with Hoechst (blue). Scale bar, 100 µm. For each panel, a representative SCO from three different experiments is shown.

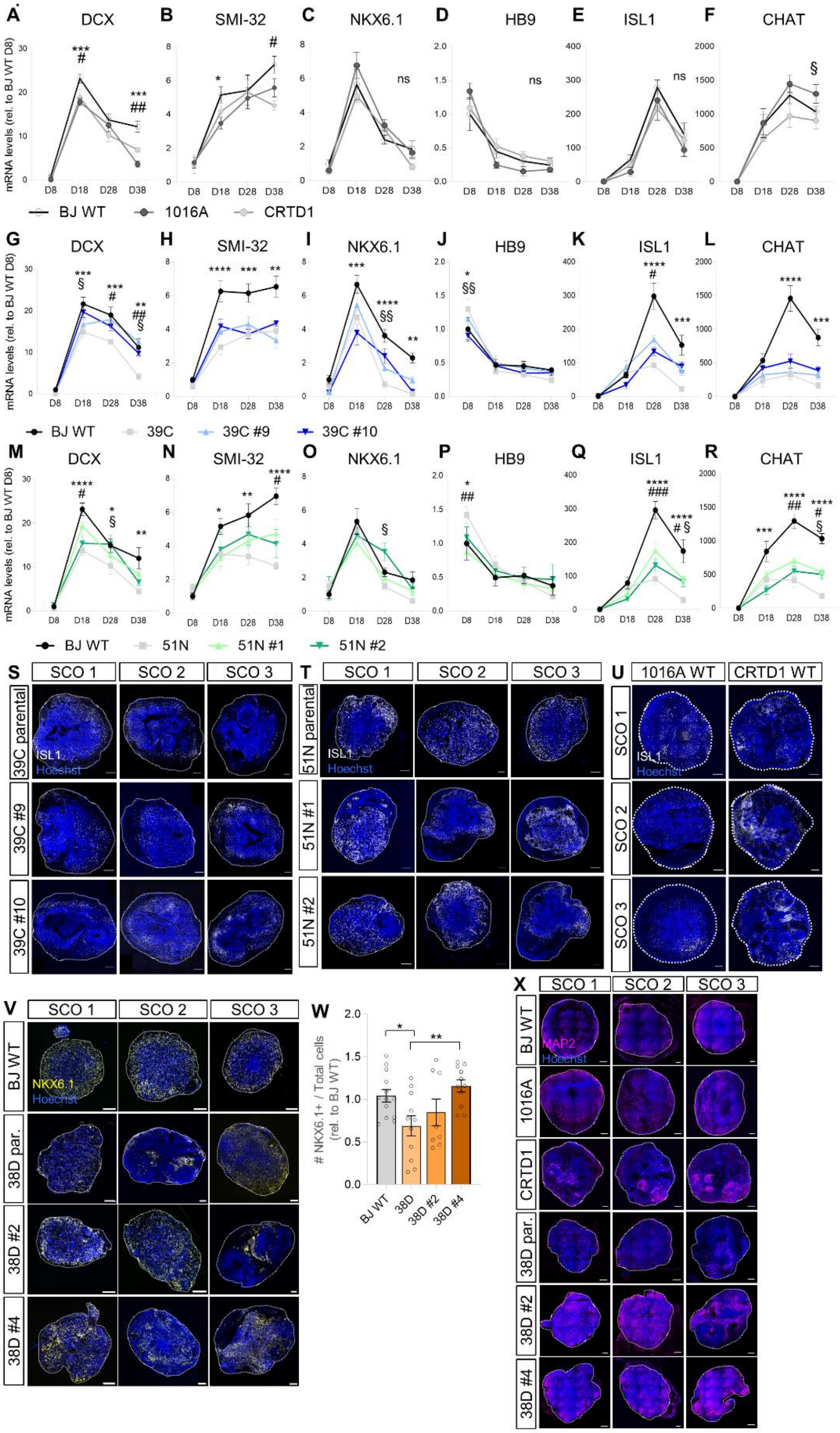

Supplemental Figure 9. **Defective neural specification programs and accelerated MN differentiation in SMA vSCOs, related to Figure 4.** mRNA qPCR quantification of *DCX*, *SMI-32*, *NKX6.1*, *HB9*, *ISL1* and *CHAT* expression in vSCOs derived from the three healthy control lines BJ, 1016A and CRTD1 (**A**-**F**), SMA 39C-III (**G**-**L**) and 51N-II isogenic trios (**M**-**R**) 8, 18, 28 and 38 days into the differentiation protocol. Gene expression is indicated as fold change of 2-ΔΔCt with respect to 18s and normalized to D8 BJ WT SCOs (Two-way ANOVA/ Fisher's LSD multiple comparison test, N=4-8, 4-8 pooled SCOs per experiment. A-F: "*" indicates comparison between BJ & 1016A; "#", comparison between BJ & CRTD1; "§",

comparison between 1016A & CRTD1. G-L: “*” indicates comparison between BJ & 39C; “#”, comparison between 39C & 39C#9; “§”, comparison between 39C & 39C#10. M-R: “*” indicates comparison between BJ & 51N; “#”, comparison between 51N & 51N#1; “§”, comparison between 51N & 51N#2). Representative images from day 28 vSCOs derived from the 39C-III (**S**) and 51N-II isogenic trios (**T**) and 1016A and CRTD1 healthy control lines (**U**) showing ISL1 (white). Nuclei stained with Hoechst (blue). Scale bar, 100 µm. (**V**) Representative images from day 28 vSCOs derived from BJ WT and the 38D-I isogenic trio showing the spinal cord ventral progenitor marker NKX6.1 (yellow). Nuclei stained with Hoechst (blue). Scale bar, 100 µm. Quantification of the percentage of NKX6.1+ cells over the total number of cells in the vSCOs (**W**). (**X**) Representative images from day 28 vSCOs derived from the 3 healthy control lines and the 38D-I isogenic trio showing the pan-neuronal marker MAP2 (magenta). Nuclei stained with Hoechst (blue). Scale bar, 100 µm. For each panel, a representative SCO from three different experiments is shown.

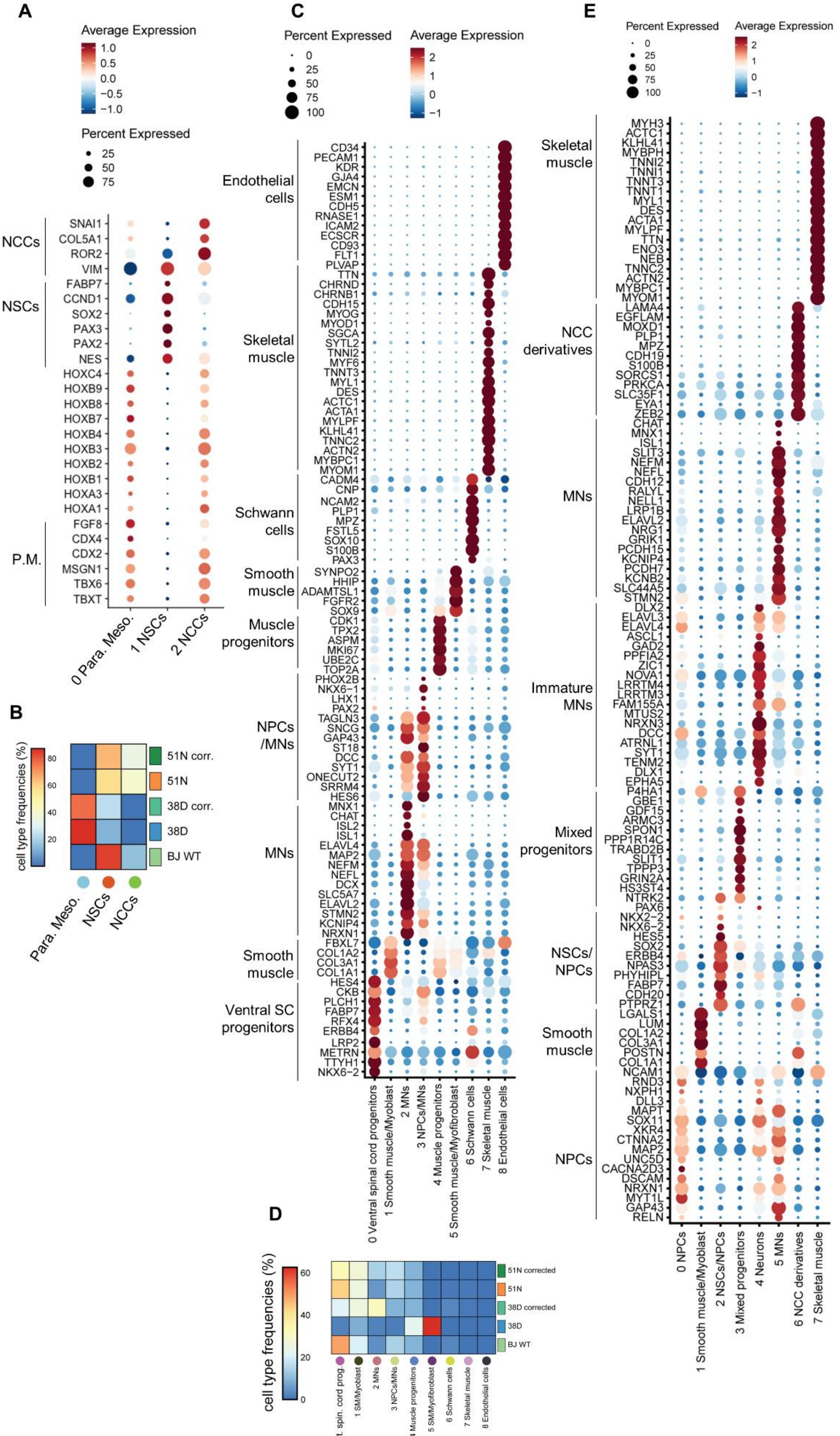
A
Average Expression
1.0
0.5
0.0
−0.5
−1.0
Percent Expressed
25
50
75
NCCs
SNAI1
COL5A1
ROR2
VIM
NSCs
FABP7
CCND1
SOX2
PAX3
PAX2
NES
HOXC4
HOXB9
HOXB8
HOXB7
HOXB4
HOXB3
HOXB2
HOXB1
HOXA3
HOXA1
P.M.
FGF8
CDX4
CDX2
MSGN1
TBX6
TBXT
0 Para. Meso.
1 NSCs
2 NCCs
B
cell type frequencies (%)
80
60
40
20
51N corr.
51N
38D corr.
38D
BJ WT
Para. Meso.
NSCs
NCCs
C
Percent Expressed
0
25
50
75
100
Average Expression
2
1
0
−1
Endothelial cells
CD34
PECAM1
KDR
GJA4
EMCN
ESM1
CDH5
RNASE1
ICAM2
ECSCR
CD93
FLT1
PLVAP
Skeletal muscle
TTN
CHRND
CHRNB1
CDH15
MYOG
MYOD1
SGCA
SYTL2
TNNI2
MYF6
TNNT3
MYL1
DES
ACTC1
ACTA1
MYLPF
KLHL41
TNNC2
ACTN2
MYBPC1
MYOM1
Schwann cells
CADM4
CNP
NCAM2
PLP1
MPZ
FSTL5
SOX10
S100B
PAX3
Smooth muscle
SYNPO2
HHIP
ADAMTSL1
FGFR2
Muscle progenitors
SOX9
CDK1
TPX2
ASPM
MKI67
UBE2C
TOP2A
NPCs /MNs
PHOX2B
NKX6−1
LHX1
PAX2
TAGLN3
SNCG
GAP43
ST18
DCC
SYT1
ONECUT2
SRRM4
HES6
MNs
MNX1
CHAT
ISL2
ISL1
ELAVL4
MAP2
NEFM
NEFL
DCX
SLC5A7
ELAVL2
STMN2
KCNIP4
NRXN1
Smooth muscle
FBXL7
COL1A2
COL3A1
COL1A1
Ventral SC progenitors
HES4
CKB
PLCH1
FABP7
RFX4
ERBB4
LRP2
METRN
TTYH1
NKX6−2
0 Ventral spinal cord progenitors
1 Smooth muscle/Myoblast
2 MNs
3 NPCs/MNs
4 Muscle progenitors
5 Smooth muscle/Myofibroblast
6 Schwann cells
7 Skeletal muscle
8 Endothelial cells
D
cell type frequencies (%)
60
40
20
0
51N corrected
51N
38D corrected
38D
BJ WT
0 Vent. spin. cord prog.
1 SM/Myoblast
2 MNs
3 NPCs/MNs
4 Muscle progenitors
5 SM/Myofibroblast
6 Schwann cells
7 Skeletal muscle
8 Endothelial cells
E
Percent Expressed
0
25
50
75
100
Average Expression
2
1
0
−1
Skeletal muscle
MYH3
ACTC1
KLHL41
MYBPH
TNNI2
TNNI1
TNNT3
TNNT1
MYL1
DES
ACTA1
MYLPF
TTN
ENO3
NEB
TNNC2
ACTN2
MYBPC1
MYOM1
NCC derivatives
LAMA4
EGFLAM
MOXD1
PLP1
MPZ
CDH19
S100B
SORCS1
PRKCA
SLC35F1
EYA1
ZEB2
MNs
CHAT
MNX1
ISL1
SLIT3
NEFM
NEFL
CDH12
RALYL
NELL1
LRP1B
ELAVL2
NRG1
GRIK1
PCDH15
KCNIP4
PCDH7
KCNB2
SLC44A5
STMN2
Immature MNs
DLX2
ELAVL3
ELAVL4
ASCL1
GAD2
PPFIA2
ZIC1
NOVA1
LRRTM4
LRRTM3
FAM155A
MTUS2
NRXN3
DCC
ATRNL1
SYT1
TENM2
DLX1
EPHA5
Mixed progenitors
P4HA1
GBE1
GDF15
ARMC3
SPON1
PPP1R14C
TRABD2B
SLIT1
TPPP3
GRIN2A
HS3ST4
NTRK2
NSCs/ NPCs
PAX6
NKX2−2
NKX6−2
HES5
SOX2
ERBB4
NPAS3
PHYHIPL
FABP7
CDH20
PTPRZ1
Smooth muscle
LGALS1
LUM
COL1A2
COL3A1
POSTN
COL1A1
NPCs
NCAM1
RND3
NXPH1
DLL3
MAPT
SOX11
XKR4
CTNNA2
MAP2
UNC5D
CACNA2D3
DSCAM
NRXN1
MYT1L
GAP43
RELN
0 NPCs
1 Smooth muscle/Myoblast
2 NSCs/NPCs
3 Mixed progenitors
4 Neurons
5 MNs
6 NCC derivatives
7 Skeletal muscle

Supplemental Figure 10. **Single-cell RNAseq cell cluster identification from day 4, 20 and 40 SCOs, related to Figures 5 and 6.** (**A**) Dot plots showing the scaled expression of selected cluster marker genes for each of the identified main cell clusters in day 4 SCOs according to Figure 5D, F. (**B**) Heatmap of cell frequencies of each cluster for BJ WT, SMA 38D- I, SMA 51N-II and their combined two isogenic controls for day 4 SCOs. (**C**) Dot plots showing the scaled expression of selected cluster marker genes for each of the identified main cell clusters in day 20 SCOs according to Figure 5E, G. The size of the dots represents the percent of cells expressing each gene, while the color depicts the scaled average expression level. (**D**) Confusion matrix of the frequencies of each cell cluster for BJ WT, SMA type I 38D, SMA type II 51N and their combined two isogenic controls at day 20 SCOs. (**E**) Dot plots showing the scaled expression of selected cluster marker genes for each of the identified main cell clusters in day 40 SCOs according to Figure 6 A, C. The size of the dots represents the percent of cells expressing each gene, while the color depicts the scaled average expression level.

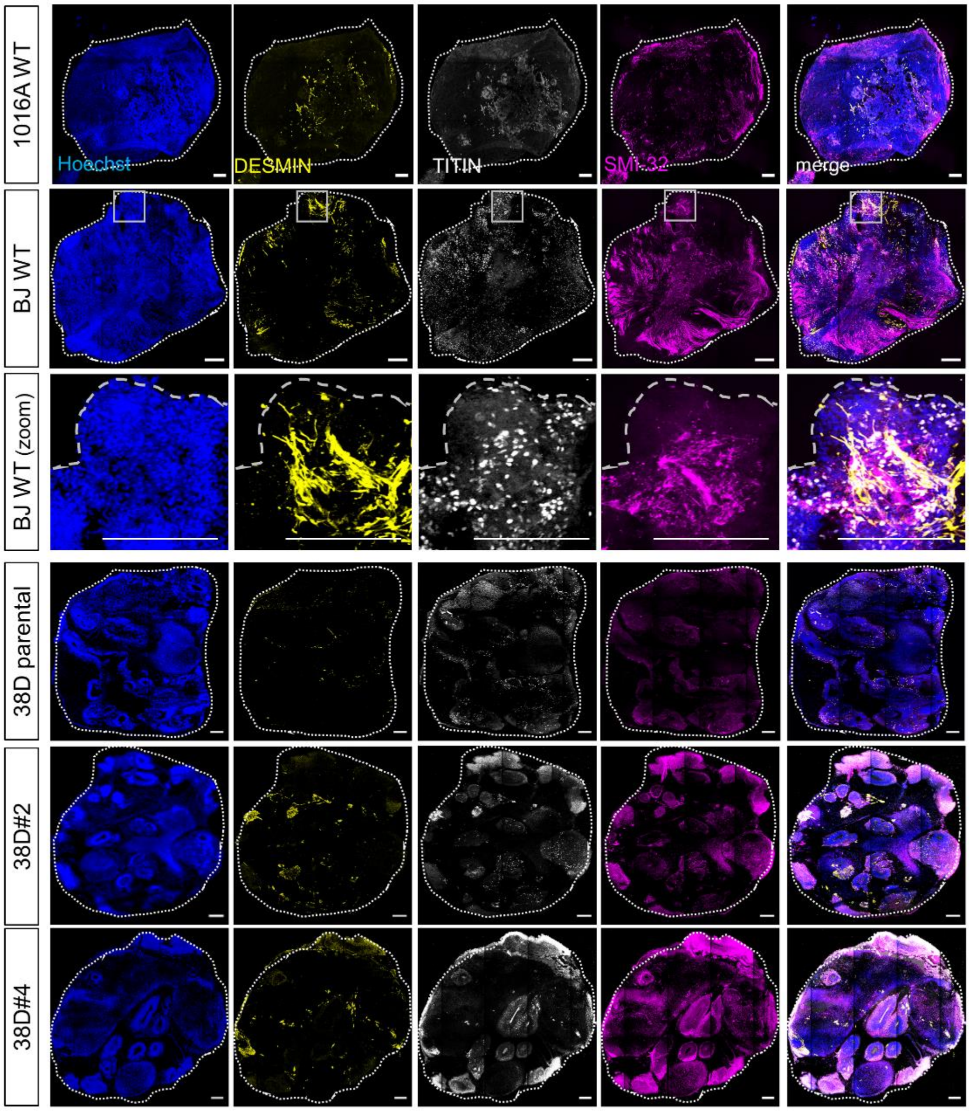

Supplemental Figure 11. **Immunostaining-based characterization of neuromesodermal organoids generated from healthy hiPSC lines and the SMA type I isogenic cohort, related to Figure 6.** Representative images of day 40 SCOs showing DESMIN (yellow), TITIN (white) (both skeletal muscle markers) and SMI-32 (magenta) (neuronal marker). Nuclei stained with Hoechst (blue). Scale bar, 100 µm. Squared regions in the BJ WT panels are shown below in magnified images (highlighted with dotted lines). Scale bar, 100 µm. One representative organoid is shown (N=3-4, 3-4 SCOs per experiment were analyzed).

Supplemental Table S1. ***SMN1-SMN2*** **genetic status of the parental hiPSC lines used in the study, related to Figure 1.**

**Human iPSCs**		***SMN1-SMN2*** **copy number analysis (MLPA)**	
Line name	Phenotype	*SMN1*	*SMN2*
BJ WT	Healthy control	2	2
1016A WT	Healthy control	2	3
CRTD1 WT	Healthy control	2	2
38D	SMA Type 1 line	0	2
51N	SMA Type 2 line	0	3
39C	SMA Type 3 line	0	3

Supplemental Table S2. ***SMN1-SMN2*** **genetic status of the genome edited hiPSC lines used in the study, related to Figure 1.** * indicates that the number of targeted *SMN2* copies in the parental type II and III lines cannot be precisely determined, 1 or 2 copies could have been converted.

	**Number of** ***SMN1*** **copies**		**Number of** ***SMN2*** **copies**	
Human iPSC line	Untargeted	Targeted (converted to *SMN1:Clover*)	Untargeted	Targeted (converted to *SMN1:Clover*)
BJ WT parental	2	-	2	-
BJ SMN:Clover # 16	2	0	1	1
BJ SMN:Clover # 23	2	0	1	1
39C parental	0	-	3	-
39C corr. #9	-	-	1*	1*
39C corr. #10	-	-	1*	1*
51N parental	0	-	3	-
51N corr. #1	-	-	1*	1*
51N corr. #2	-	-	1*	1*
38D parental	0	-	2	-
38D corr. #2	-	-	1	1
38D corr. #4	-	-	1	1

Supplemental Table S3. gRNAs and primers used, related to STAR Methods. gRNAs used for the generation of the isogenic hiPSC lines (top), primers used for PCR-amplification of *SMN* loci followed by Sanger sequencing (middle) and primers used for qPCR mRNA expression quantification for hiPSC Trilineage differentiation assay (genes recommended by STEMdiff Trilineage Differentiation Kit), for neural differentiation of vSCOs and snRNA analysis (bottom).

**gRNA used to generate *SMN1:Clover* hiPSC lines**			
**gRNA-1 FOR (5´→ 3´)**	CACCTGCTCACATTCCTTAAATTA		
**gRNA-1 REV (5´→ 3´)**	AAACTAATTTAAGGAATGTGAGCA		
**RT-PCR analysis of *SMN* loci followed by Sanger Sequencing**			
	**Target Gene**	**Sequence forward (5´→ 3´)**	**Sequence reverse (5´→ 3´)**
**Detection of non-targeted gene**	*SMN2*	GTTCTCCAAATCCGACCTCA	TTTCTTCCACATAACCAACCAG
**Targeted (SMN2 to SMN1 conversion)**	*SMN2*	GCCCGGCCTAGTCTTGTATT	AGGTACCGTCGTCCTTGAAA
**Targeted (SMN1:Clover)**	*SMN2*	CAGGAGGATTCCGTGCTGTT	AGGTACCGTCGTCCTTGAAA
**RT-qPCR analysis**			
	**Target Gene**	**Sequence forward (5´→ 3´)**	**Sequence reverse (5´→ 3´)**
**hiPSC trilineage differentiation**	*SOX1*	CTGACGTCCACTCTCAGTCT	CCACATCCTAATCTTGAGCCA
	PAX6	TTGCCCGAGAAAGACTAGCA	TGGAGCCAGATGTGAAGGAG
	NCAM1	GACCATCCACCTCAAAGTCTT	GAGGCTTCACAGGTAAGAGTG
	TBXT	CCACATAGTGAGAGTTGGGG	AGAGCTGTGATCTCCTCGT
	CXCR4	AAATCTTCCTGCCCACCATC	GTACTTGTCCGTCATGCTTCT
	LEFTI1	CTTGGGGACTATGGAGCTCAGG	ATGTACATCTCCTGGCGGC
	SOX17	AACGCCGAGTTGAGCAA	GGCCGGTACTTGTAGTTGG
**SCO developmental gene expression**	*SOX2*	GTACAACTCCATGACCAGCTC	CTTCAGCACCGAACCCAT
	NESTIN	CTCAGCTTTCAGGACCCCAAG	TCTCAAGGGTAGCAGGCAAG
	NGN2	GCCAAAGTCACAGCAACG	TCCTCTTCCTCCTTCAACTCC
	DCX	GTGTTTATTGCCTGTGGTCCTG	GGAGGTTCCGTTTGCTGAGT
	SMI-32	GAGTGGTTCCGAGTGAGGCTG	AGTGAGTCCTTGGTGCTTTTCAG
	NKX6.1	CCTGTACCCCTCATCAAGGA	GAATAGGCCAAACGAGCCCT
	HB9	CTGGAGCACCAGTTCAAGCTCA	TGGAACCAAATCTTCACCTGGGT
	ISL1	TGCTTTTCAGCAACTGGTCAAT	AGGACTGGCTACCATGCTGT
	CHAT	CGACAAGTCCCTGCATTTG	ACGGAGTCTGCTCGGATCA
**Housekeeping gene**	*18S*	AAACGGCTACCACATCCAAG	CCTCCAATGGATCCTCCATA
**Spliceosome snRNAs measurement**	*U1*	GATACCATGATCACGAAGGTGGTT	CACAAATTATGCAGTCGAGTTTCC
	U4	GCGCGATTATTGCTAATTGAAA	AAAAATTGCCAATGCCGACTA
	U5	GGTTTCTCTTCAGATCGCATAAATC	CTCAAAAAATTGGGTTAAGACTCAGA
	U6	GCTTCGGCAGCACATATACTAAAAT	ACGAATTTGCGTGTCATCCTT
	U11	GTGCGGAATCGACATCAAGAG	CGCCGGGACCAACGAT
	U12	AACTTATGAGTAAGGAAAATAACGATTCG	CGACCTTTACCCGCTCAAAA
	U4atac	GCGCATAGTGAGGGCAGTACT	GCACCAAAATAAAGCAAAAGCTCTA
	U6atac	AGGTTAGCACTCCCCTTGACAA	TGGCAATGCCTTAACCGTATG
	5.8 S	CGGCTCGTGCGTCGAT	CCGCAAGTGCGTTCGAA
